# Chromosome-Scale, De Novo, Phased Genome Assemblies of Three Australian Limes: *Citrus australasica*, *C. inodora*, and *C. glauca*

**DOI:** 10.3390/plants13111460

**Published:** 2024-05-24

**Authors:** Khushwant Singh, Matthew Huff, Jianyang Liu, Jong-Won Park, Tara Rickman, Manjunath Keremane, Robert R. Krueger, Madhurababu Kunta, Mikeal L. Roose, Chris Dardick, Margaret Staton, Chandrika Ramadugu

**Affiliations:** 1 Department of Botany and Plant Sciences, University of California, Riverside, CA 92521, USA; khushwas@ucr.edu (K.S.); mikeal.roose@ucr.edu (M.L.R.); 2 Department of Entomology and Plant Pathology, University of Tennessee, Knoxville, TN 37996, USA; huffmat@musc.edu (M.H.); tara@phasegenomics.com (T.R.); mstaton1@utk.edu (M.S.); 3 Innovative Fruit Production, Improvement, and Protection, Appalachian Fruit Research Station, USDA-ARS, Kearneysville, WV 25430, USA; jianyang.liu@usda.gov (J.L.); chris.dardick@usda.gov (C.D.); 4 Citrus Center, Texas A&M University-Kingsville, Weslaco, TX 78599, USA; jongwon.park@tamuk.edu (J.-W.P.); madhura.kunta@tamuk.edu (M.K.); 5 National Clonal Germplasm Repository for Citrus and Dates, USDA-ARS, Riverside, CA 92507, USA; manjunath.keremane@usda.gov (M.K.); robert.krueger@usda.gov (R.R.K.)

**Keywords:** citrus, huanglongbing, Australian limes, phased genomes, *C. australasica*, *C. inodora*, *C. glauca*

## Abstract

Huanglongbing (HLB) is a severe citrus disease worldwide. Wild Australian limes like *Citrus australasica*, *C. inodora*, and *C. glauca* possess beneficial HLB resistance traits. Individual trees of the three taxa were extensively used in a breeding program for over a decade to introgress resistance traits into commercial-quality citrus germplasm. We generated high-quality, phased, de novo genome assemblies of the three Australian limes using PacBio long-read sequencing. The genome assembly sizes of the primary and alternate haplotypes were determined for *C. australasica* (337 Mb/335 Mb), *C. inodora* (304 Mb/299 Mb), and *C. glauca* (376 Mb/379 Mb). The nine chromosome-scale scaffolds included 86–91% of the genome sequences generated. The integrity and completeness of the assembled genomes were estimated to be at 97.2–98.8%. Gene annotation studies identified 25,461 genes in *C. australasica*, 27,665 in *C. inodora*, and 30,067 in *C. glauca*. Genes belonging to 118 orthogroups were specific to Australian lime genomes compared to other citrus genomes analyzed. Significantly fewer canonical resistance (*R*) genes were found in *C. inodora* and *C. glauca* (319 and 449, respectively) compared to *C. australasica* (576), *C. clementina* (579), and *C. sinensis* (651). Similar patterns were observed for other gene families associated with potential HLB resistance, including Phloem protein 2 (*PP2*) and Callose synthase (*CalS*) genes predicted in the Australian lime genomes. The genomic information on Australian limes developed in the present study will help understand the genetic basis of HLB resistance.

## 1. Introduction

The citrus industry in the western hemisphere has been seriously affected by huanglongbing (HLB) since 2004. The disease is associated with an unculturable alpha-proteobacterium, *Candidatus* Liberibacter asiaticus (CLas) [1,2]. Cultivated citrus varieties do not have resistance to HLB; tolerance to different levels is reported in some cultivars. In a long-term field experiment conducted in an HLB-endemic region of Florida, we determined that the Australian limes possess resistance and field tolerance to HLB [3]. This finding was corroborated by other researchers in greenhouse studies [4,5,6]. In the field conditions of an HLB-endemic region in Florida, the Australian limes *C. australasica*, *C. inodora*, and *C. glauca* exhibited different disease tolerance/resistance levels. We have had a breeding program since 2013 focused on transferring HLB resistance or tolerance from Australian limes to citrus. We produced approximately 4000 novel hybrids (first-generation F1 and second-generation advanced hybrids) with various HLB resistance/tolerance levels. Our greenhouse and field experiments showed that the progeny in our breeding population can inherit the disease resistance observed in the Australian limes. Identifying the target genes/QTLs associated with HLB resistance will be very useful in introducing the resistance traits into commercial citrus varieties through biotechnological methods like plant transformation, gene editing, etc. In addition to the identification of HLB-resistant hybrids, characterization of the genetic basis of disease resistance may help identify the candidate genes for conferring resistance in existing commercial citrus.

Unlike clonally propagated citrus cultivars, the wild Australian limes are generally seed-propagated, and our knowledge of their genetic diversity is limited. Here, we report the genomic sequences of three individual trees: *C. australasica* (inventory number, IVNO 6502)*, C. inodora* (IVNO 2781), and *C. glauca* (IVNO 6206). Our ongoing breeding program selected these trees as HLB-resistant/tolerant sources based on our field studies [3,7,8]. These three trees are maintained at the Givaudan Citrus Variety Collection (GCVC) of the University of California Riverside (UCR). Further information on accessions is available from UCR and the United States Department of Agriculture (USDA; Supplementary Table S1). The genetic materials (seeds, nucleic acids, etc.) from all three trees are available from The National Clonal Germplasm Repository (NCGR, USDA), Riverside, CA, USA. Sanitized budwood will be available in the future.

The taxonomic identification of Australian finger lime, *C. australasica* F. Muell. [9], was changed to *Microcitrus australasica* (F. Muell.) Swing. in 1915 [10]; the Russell River lime, *C. inodora* Bail. [11], was changed to *M. inodora* [10]; the Australian desert lime, *Eremocitrus glauca* (Lindley) Swing. [12], was changed to *C. glauca* (Lindley) Burkill [13]. In 1998, Mabberley placed all three taxa under the genus *Citrus*, primarily based on the availability of hybrids with other *Citrus* types [14].

Present-day citrus cultivars originated in China, India, and Myanmar, which are land-connected [15,16]. The genetic diversity within cultivated citrus is limited because of the extensive clonal propagation of a small number of varieties. While finding the sources of HLB resistance in wild relatives in Asia is theoretically possible, such exploratory findings are rare. Australian limes were geographically isolated from other citrus types for several million years [15]. The mechanism of resistance/tolerance against CLas found in Australian limes may differ from that of cultivated citrus.

To investigate the unique features of Australian limes, including *C. australasica*, *C. inodora*, and *C. glauca*, we performed long-read genome sequencing and assembled de novo phased genomes. We investigated the gene family evolution by conducting a comparative genomic analysis of the three Australian lime genomes and reference genomes, such as *C. clementina*, *C. sinensis*, and *C. trifoliata* (synonym of *Poncirus trifoliata*). These high-quality genome assemblies unveiled the features and evolutionary insights for the three Australian lime genomes. They will serve as essential resources for genetic, genomic, and molecular research and facilitate the development of HLB resistance in citrus. From an economic perspective, the availability of HLB-resistant/tolerant citrus hybrids that have introgressed resistance-associated genes is extremely valuable; hybrids that also possess acceptable organoleptic fruit attributes would rejuvenate the citrus industries in regions ravaged by the HLB disease.

## 2. Results

### 2.1. Genome Sequencing and De Novo Assembly of Australian Limes

**Figure 1:**
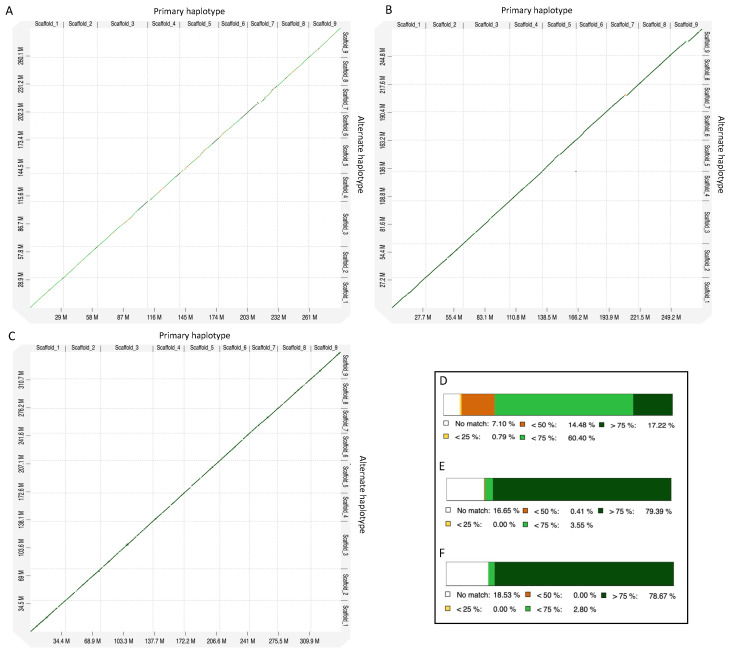
Dot plot comparison of primary vs. alternate haplotypes of (**A**) *Citrus australasica*, (**B**) *C. inodora*, and (**C**) *C. glauca*. The bottom right panel shows the percent identity between the primary and alternate haplotypes of (**D**) *C. australasica*, (**E**) *C. inodora*, and (**F**) *C. glauca*.

De novo haplotype assemblies of the three Australian limes (Figure 1, Supplementary Table S1) using PacBio and Hi-C resulted in chromosome-scale scaffolds (Table 1, Supplementary Figure S1). The total number of scaffolds was 83 in *Citrus glauca*, 270 in *C. australasica*, and 299 in *C. inodora* (Table 1). Approximately 86–91.5% of the bases were assembled into nine pairs of chromosomes with N50 values ranging from 29 Mb to 37 Mb (Table 1). The genome sizes ranged from 380 Mb in *C. glauca* to 335 Mb in *C. australasica* and 300 Mb in *C. inodora*. The GC content in the three genomes ranged from 36–37% (Table 1). In all three genomes, the nine chromosomal pseudomolecules were numbered and oriented according to the reference genomes of *C. clementina* and *C. trifoliata* (Supplementary Table S2). Chromosome 3 was the largest among all three Australian lime species: 59 Mb in *C. glauca*, 47 Mb in *C. australasica*, and 42 Mb in *C. inodora*. Individual chromosome-scale scaffold sizes are provided in Supplementary Table S2.

The comparison of de novo assembled genomes in the dot plot graphs shows that the nine chromosome-scale scaffolds of primary and alternate haplotypes align well in all three genomes (Figure 1A–C), with no inverted repeat sequences or homopolymeric regions (low complexity regions, LCRs). Primary and alternate haplotypes exhibited approximately 84% similarity among the three genomes (Figure 1D–F).

The completeness of the genomes and the predicted protein sequences were evaluated using BUSCO scores. The genome assembly completeness was highest in *C. australasica* (98.70–99.20%), followed by *C. glauca* (96.90–97.20%) and *C. inodora* (96.68–96.70%) (Table 2); over 97% of the long reads aligned with the final assemblies of the respective genomes. For the predicted protein sequences, the completeness was highest in *C. australasica* (96.80–99.10%), followed by *C. glauca* (94.50–97.40%) and *C. inodora* (92.80–94.60%) (Table 2).

### 2.2. Genomic Variation between the Primary and Alternate Haplotypes in the Three Australian Limes

**Figure 2:**
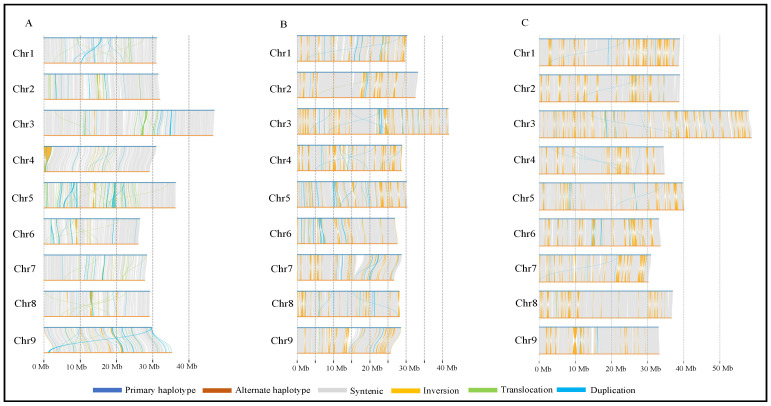
Syntenic relationship between primary and alternate haplotypes of (**A**) *Citrus australasica*, (**B**) *C. inodora*, and (**C**) *C. glauca*. The haplotype assemblies of de novo genomes were mapped using Synteny and Rearrangement Identifier (SyRI). Synteny relationship analysis between primary and alternate haplotypes was performed using minimap2 and samtools. Graphical representation was created using Plotsr (https://github.com/schneebergerlab/plotsr, accessed on 19 June 2023).

The genomic variation between the haplotypes was investigated based on a Synteny and Rearrangement Identifier (SyRI) analysis (Figure 2). Overall, across the three Australian limes, both the primary and alternate haplotypes exhibited variations of less than 0.1%, including single-nucleotide polymorphisms (SNPs), insertions, deletions, translocations, duplications, and inversions (Table 3). The total genomic variations between the primary and alternate haplotypes fell within a range of 2.30 Mb in *C. australasica*, 2.92 Mb in *C. inodora*, and 2.90 Mb in *C. glauca*, as illustrated in Figure 2 and Table 3. Among all variations observed in the three Australian lime genomes, SNPs were predominant compared to other structural variations (SVs) (Supplementary Table S3).

### 2.3. Syntenic Relationships of the Three Australian Lime Genomes with Reference Genomes

**Figure 3:**
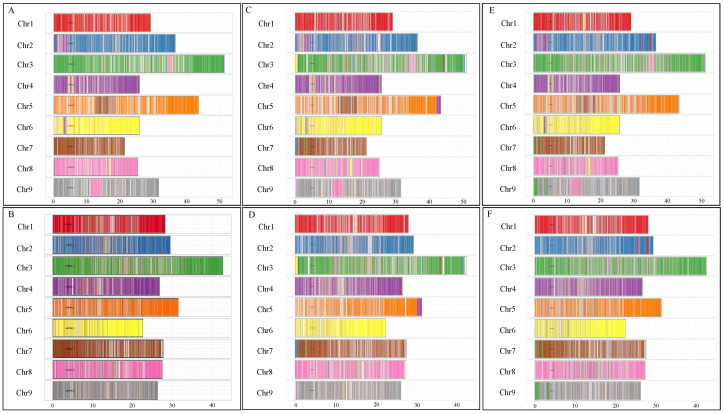
Mapping of Australian lime genomes on *Citrus clementina* and *C. trifoliata* reference genomes. Primary haplotype sequences from (**A**) *C. australasica*, (**C**) *C. inodora*, and (**E**) *C. glauca* were used as query sequences, while the target sequence (*x*-axis) represents the chromosomes of *C. clementina*. Primary haplotype sequences from (**B**) *C. australasica*, (**D**) *C. inodora*, and (**F**) *C. glauca* were used as query sequences; the target sequence (*x*-axis) represents the chromosomes of *C. trifoliata*. Chromosome scale positions are shown in Mb. Final plots were constructed using the Pafr and ggplots packages in open-source Rstudio (R Core Team, 2021).

Genome coverage plots were constructed after mapping the Australian lime genomes with *C. clementina* (Figure 3A,C,E) and *C. trifoliata* (Figure 3B,D,F) using minimap2. The *C. clementina* genome showed more significant structural differences upon mapping with all three Australian limes (Figure 3 and Supplementary Figure S2). For example, chromosome 2 has a 3.5 Mb region inserted at position 0.5 Mb–5 Mb of chromosome 4 (highlighted in purple); chromosome 3 has a region of 1 Mb (highlighted in pink) from chromosome 8 at 34–35 Mb; chromosome 5 has a region of 6 Mb (highlighted in brown) from chromosome 7 at position 12–18 Mb; chromosome 6 has a region of less than 0.25 Mb (highlighted in purple) from chromosome 4 at position 5 Mb; chromosome 8 has a region of less than 0.1 Mb (highlighted in yellow) from chromosome 6 at position 17.5 Mb; and chromosome 9 has a region greater than 4.5 Mb (highlighted in pink) from chromosome 8 at position 11–15 Mb (Figure 3A,C,E and Supplementary Figure S2).

The *C. inodora* genome showed unique additional patterns compared to both the reference genomes. For example, chromosome 3 has multiple regions inserted from chromosomes 1, 2, 6, and 8; chromosome 5 has a region of approximately 1 Mb (highlighted in purple) from chromosome 4; chromosome 7 has inserts from chromosomes 2 and 3; and chromosome 9 has an insert from chromosome 4 (Figure 3C,D and Supplementary Figure S2). Additional unique patterns were found in *C. glauca* compared to the reference genomes. For example, chromosome 1 has a fragment inserted from chromosome 6 (highlighted in yellow); chromosome 2 has a fragment inserted from chromosome 1 (highlighted in red); chromosomes 7 and 9 have a region inserted from chromosome 3 (highlighted in green) (Figure 3E,F and Supplementary Figure S2).

### 2.4. Divergence of Australian Lime Genomes Compared with Reference Genomes

The approximate per-base divergence between primary and alternate haplotypes showed low divergence in *C. inodora* (0.034–0.052 per base), followed by *C. glauca* (0.042–0.054 per base) and *C. australasica* (0.052–0.061 per base) (Supplementary Figure S3A). Per-base divergence of the primary and alternate haplotypes of Australian limes with the *C. clementina* genome showed that *C. inodora* is less divergent (0.071–0.08 per base) compared to *C. australasica* (0.079–0.085 per base) and *C. glauca* (0.082–0.096 per base) (Supplementary Figure S3B). Compared with *C. trifoliata*, per-base divergence showed similar numbers in *C. australasica* and *C. inodora* (0.87–0.95 per base), while *C. glauca* showed higher divergence at 0.97–0.112 per base (Supplementary Figure S3C).

### 2.5. Gene Annotations of Australian Lime Genomes

**Figure 4:**
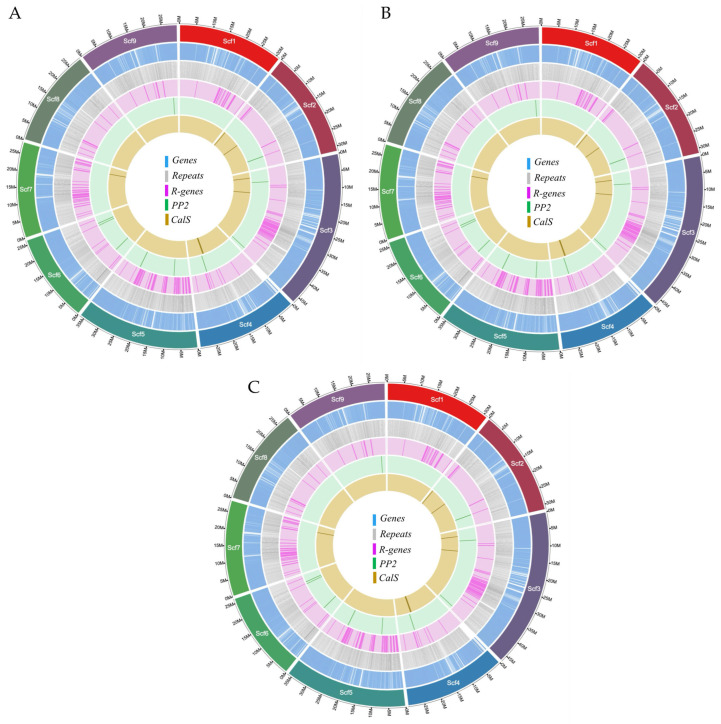
Characterization of the primary haplotypes of the three genomes of (**A**) *C. australasica*, (**B**) *C. inodora*, and (**C**) *C. glauca*. From outer to inner rings: I. The nine assembled chromosome-scale scaffolds (in Mb) correspond to the nine chromosomes (Scf1−Scf9) of *Citrus clementina*; II. Locations of the predicted gene models; III. Long terminal repeat (LTR) transposable elements (TEs); IV. Nucleotide-binding site (NBS)-containing genes (*R* genes); V. Phloem protein 2 (*PP2)* genes; and VI. Callose synthase (*CalS*) genes. Circa was used to draw the circos plots.

Through de novo repeat discovery and a search for known repetitive elements, our pipeline identified approximately 55% repetitive content in *C. australasica*, 44% in *C. inodora*, and 51% in *C. glauca*. Long terminal repeats (LTRs) were the most identified class in all the assemblies, ranging from 21 to 23%. The abundance of LTRs in all three assemblies is consistent with previously assembled plant genomes [18,19]. We produced an initial set of high-confidence gene models for each assembly and then performed structural filtering. The final gene models varied between haplotypes and species. *C. australasica* had the lowest number of predicted genes, with 27,358 in the primary and 25,461 in the alternate haplotype, followed by *C. inodora*, with 28,176 in the primary and 27,665 in the alternate haplotype. *C. glauca* had the most genes, with 30,067 in the primary and 33,673 in the alternate haplotypes (Table 4). Of these models, 73–90% of genes per haplotype were annotated with either a sequence similarity match to a known protein database or assignment to an eggNOG gene family (Table 4). In addition, in all three Australian limes, the annotated genes were well distributed throughout the chromosome-scale scaffolds in the nine large chromosomes in both the primary and alternate haplotypes, as depicted in Figure 4 and Supplementary Figure S4.

OrthoFinder analysis of nine citrus species placed 244,631 genes out of 257,276 into 27,932 orthogroups. The remaining 12,645 genes were not placed, indicating singletons with no known orthologs in other species. Most genes were placed into orthogroups for each species (Supplementary Table S4). We identified 118 orthogroups containing at least one gene from all three Australian lime species and none from other citrus species. Most of the genes from all three Australian limes were placed in orthogroups with other citrus species; a small number were placed in species-specific orthogroups or considered singletons. We calculated the number of species-specific and unplaced genes for each Australian lime species. We reported 1372 genes in 91 orthogroups specific to *C. australasica*, 286 genes specific to 105 orthogroups in *C. inodora*, and 1421 genes in 155 orthogroups specific to *C. glauca* (Supplementary Table S4). GO enrichment analysis revealed that genes within the 118 orthogroups shared by the three Australian lime species but absent in other citrus exhibited association with methylation, phosphorous metabolism, peptidase activity, and anion bindings (Supplementary Figures S5–S7).

**Figure 5:**
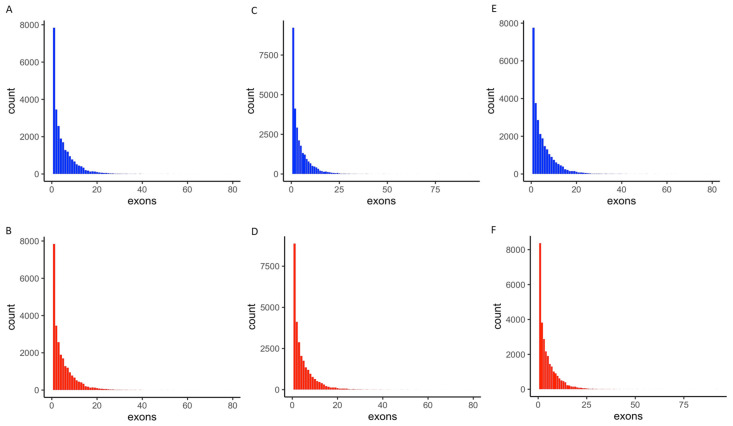
Transcript distribution based on the number of exons in *C. australasica* primary (**A**) and alternate haplotypes (**B**), *C. inodora* primary (**C**) and alternate haplotypes (**D**), and *C. glauca* primary (**E**) and alternate haplotypes (**F**). The scaled positions were drawn using the ggplots package in open-source Rstudio.

Exons per transcript were analyzed in the nine large chromosome-scale scaffolds in the primary and alternate haplotypes (Figure 5). More than 99% of the transcripts in all three taxa contained more than one exon. Transcripts with two exons were abundant (28.12–40.25%), followed by three exons (12.0–15%), four (9–11%), and five (7–8%) (Figure 5). *C. inodora* did not show differences between the primary and alternate haplotypes regarding the number of exons per transcript. *C. australasica* and *C. glauca* showed variation between the primary and alternate haplotype transcript exon numbers. For example, in *C. australasica*, transcripts with two exons accounted for 33% in the primary and 28% in the alternate haplotype, and transcripts with three exons accounted for 14.0% in the primary haplotype and 15.0% in the alternate haplotype. In *C. glauca*, transcripts with two exons in the primary and alternate haplotypes accounted for 34.0% and 41.0%. Transcripts with three exons constituted 13.0% in the primary and 12.0% in the alternate haplotypes. Transcripts containing four exons accounted for 10.0% in the primary and 9.0% in the alternate haplotypes.

Serine/threonine-protein kinase is the largest predicted transcript at 9 kb with 80 exons in all three genomes. This highly conserved gene is localized on chromosome 3 in all three taxa. The serine/threonine-protein kinase consists of a Phosphatidylinositol 3- and 4-kinase (PI3_PI4 kinase), FAT domain, FATC domain, and Tetratricopeptide repeat (Supplementary Figure S8A). An HMM logo of the transcript is presented in Supplementary Figure S8B.

### 2.6. Identification and Classification of NBS-Based R Genes in Five Citrus Species

The number of *R* genes that had both nucleotide-binding sites (NBS) and leucine-rich repeat (LRR) domains (NLR) ranged from 319 to 651 in the three Australian lime genomes and two domesticated citrus cultivars (Table 5). The highest number of *R* genes was found in *C. sinensis*, followed by *C. clementina*, with *C. inodora* having the lowest number of *R* genes. In each of the three Australian lime genomes, more than 50% of the total *R* genes fall in either the CNL cluster [NLRs with coiled-coil (CC) N-terminal domain] or the TNL (NLRs with Toll/Interleukin-1 receptor) category, with the former being more prevalent than the latter. In all three species, the number of *R* genes accounts for less than 2.4% of the total genes in the genome. The location of the *R* genes in the nine large chromosome-scale scaffolds is shown in Figure 4 and Supplementary Figure S4. Most of the *R* genes are clustered on chromosome 5 (27–33%), chromosome 3 (26–31%), and chromosome 7 (15–17%). The primary haplotype of *C. australasica* contained 195 *R* genes on chromosome 5, while the alternate haplotype had 150 *R* genes. *C. inodora* contained only 88 *R* genes on chromosome 5. Chromosome 6 of *C. australasica* and *C. inodora* contained the lowest number of *R* genes (4–10). In *C. glauca*, chromosome 4 had only six *R* genes in each haplotype (Figure 4 and Supplementary Figure S4).

### 2.7. PP2 and Callose Synthase Gene Family Analysis

**Figure 6:**
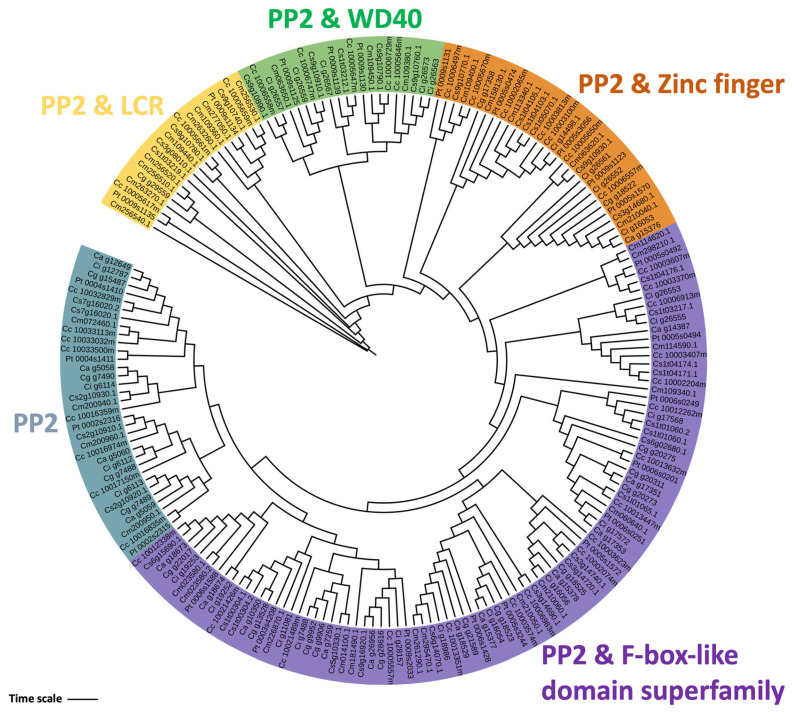
Phylogenetic tree depicting the evolutionary relationship between PP2 from *C. australasica* (Ca), *C. inodora* (Ci), *C. glauca* (Cg), *C. sinensis* (Cs), *C. clementina* (Cc), and *C*. *medica* (Cm), *C. trifoliata* (Pt). Protein sequences were aligned using ClustalX2 to generate the phylogenetic tree, and the tree was constructed using MEGA X and viewed with iTOL. The maximum-likelihood method was used for the construction of the tree, and the reliability of the branches was inferred from a bootstrap analysis of 1000 replicates.

To investigate genes encoding Phloem protein 2 (PP2) (EC 3.2.2.6) and Callose synthase (CalS) (EC 2.4.1.34), the protein profiles from the PFAM database were mapped using HMM. Overall, *C. australasica* had 47 *PP2*-protein-encoding genes, *C. inodora* contained 51 *PP2*-encoding genes, and *C. glauca* had the highest number, with 56 *PP2*-protein-encoding genes (Table 6). The chromosome localization of *PP2* is shown in Figure 4. Interestingly, in all three Australian lime genomes, *PP2*-protein-encoding genes were not found on chromosomes 1, 7, and 8 (Figure 4). *PP2*-protein-encoding genes in chromosome 9 were clustered in both haplotypes in *C. inodora* but in only one haplotype in both *C. australasica* and *C. glauca* (Figure 4). In all three Australian limes, chromosome 9 contained over 11 *PP2*-protein-encoding genes (Supplementary Figure S4). In the primary haplotype of *C. inodora*, chromosome 9 showed nine *PP2*-protein-encoding genes (Figure 4). Phylogenetic analysis of *PP2* from the three Australian limes, other citrus types (*C. clementina*, *C. sinensis*, and *C. medica*), and *C. trifoliata* grouped the genes into five clusters (Figure 6). All five clusters contained *PP2* genes with single or multi-catalytic domains. The additional domains found included the low complexity region (LCR) in cluster I, the WD40 domain in cluster II, the Zinc finger domain in cluster III, the F-box-like domain superfamily in cluster IV, and the PP2 domain in cluster V (Figure 6).

*CalS*-protein-encoding genes fell within a range of 22 in *C. australasica*, 20 in *C. inodora*, and 19 in *C. glauca* (Table 6). The chromosome localization of *PP2* genes is shown in Figure 4. In all three taxa, *CalS*-encoding genes were not found on chromosomes 1, 5, 8, and 9, while chromosome 6 also lacked the *CalS* gene in all species except *C. inodora* (Figure 4 and Supplementary Figure S4). Phylogenetic analysis of *CalS* genes from the three Australian limes and citrus types studied did not show clustering as was observed with *PP2* genes.

### 2.8. Analysis of RNA-Seq Data

We conducted a transcriptome analysis of *C. australasica*, *C. inodora*, and *C. glauca* to improve the annotation of the genome assemblies. RNA-seq was performed with total RNA from various tissues (Supplementary Table S5). Eight samples of *C. australasica* yielded 67–81 million (M) paired-end reads; six samples of *C. inodora* and *C. glauca* yielded 65–72 M reads and 74–82 M reads, respectively. More than 88% of the bases had quality scores of Q ≥ 30 (Supplementary Table S5).

For the discovery of novel and transcript isoforms, quality-filtered RNA-seq reads obtained from various tissue samples of each Australian lime species (Supplementary Tables S5 and S6) were pooled and mapped to the corresponding genome assemblies (primary and alternate haplotypes) using the “Large Gap Read Mapping” tool in CLC genomics workbench version 24.0.1 (Qiagen, San Diego, CA, USA). The approximate number of reads mapped to the assemblies were 80.7% for *C. australasica*, 93% for *C. inodora*, and 94.3% for *C. glauca* (Table 7). According to the gene models for the genomes of the three Australian lime species, the predicted number of transcripts was 1.1 per gene (Supplementary Table S7).

De novo transcriptome assembly with *C. australasica* reads generated 252,646 contigs with an average length of 1985 bp and N50 contig length of 3641 bp. With *C. inodora* reads, 282,079 contigs were obtained with an average length of 1948 bp and N50 contig length of 3772 bp. With *C. glauca* reads, we generated 283,961 contigs with an average length of 1832 bp and N50 contig length of 3654 bp (Supplementary Table S6). The BUSCO scores of the de novo transcriptome assemblies were 93.7% for *C. australasica*, 93.4% for *C. inodora*, and 95.3% for *C. glauca* (Supplementary Table S8).

Before functional annotation of the de novo transcriptome assemblies, the redundant sequences from the de novo assemblies of *C. australasica*, *C. inodora*, and *C. glauca* were removed by the CD-HIT clustering tool in OmicsBox v3.0.30, from which a total of 186,986 contigs for *C. australasica*, 217,158 contigs for *C. inodora*, and 221,159 contigs for *C. glauca* were obtained (Supplementary Table S9). The number of predicted open reading frames [ORFs by TransDecoder (v5.5.0)] in these contigs were 140,778 for *C. australasica*, 166,765 for *C. inodora*, and 149,885 for *C. glauca* (Supplementary Table S9). Approximately 62–65% of the predicted ORFs were functionally annotated (Supplementary Table S9).

**Figure 7:**
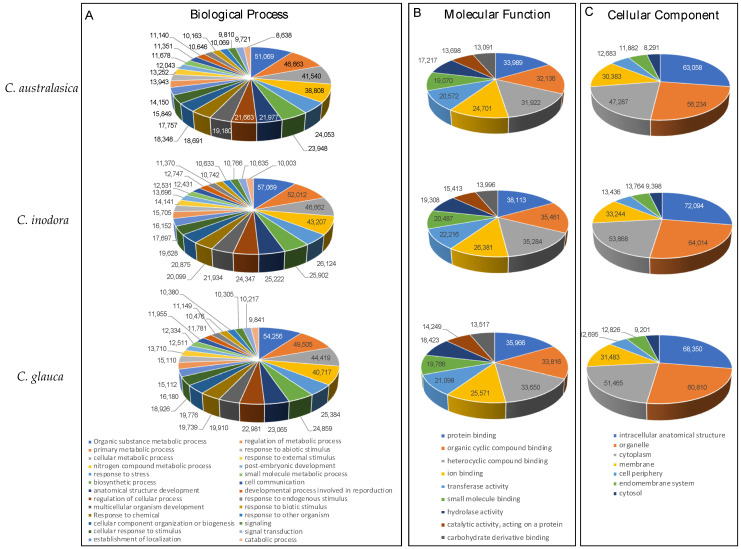
Gene Ontology (GO) classification of annotated sequences of de novo transcriptome assemblies of the three Australian lime species at Level 3 of three GO categories: Biological Process (**A**), Molecular Function (**B**), and Cellular Component (**C**). The name of each species is indicated in the figure. The color-coded GO terms are listed at the bottom.

Among the three main GO categories (i.e., Biological Process, Cellular Component, and Molecular Function), 81,000–90,000 sequences were assigned to Biological Process (GO:0008150), 77,000–87,000 sequences to Cellular Component (GO:0005527), and 81,000–91,000 sequences to Molecular Function (GO:0003674) (Supplementary Table S10). The analysis of GO terms of the annotated sequences at Level 3 of the three GO categories revealed that the most enriched, top four GO terms in each category were (1) in biological process, organic substance metabolic process, primary metabolic process, cellular metabolic process, and nitrogen compound metabolic process; (2) in molecular function, protein binding, organic cyclic compound binding, heterocyclic compound binding, and ion binding; and (3) in cellular component, intracellular anatomical structure, organelle, cytoplasm, and membrane categories (Figure 7).

Among the 81,000–90,000 sequences involved in Biological Processes (Supplementary Table S10), 11.8–12.4% are predicted to be involved in the response to other organisms (GO:0009725), of which 81–83% are presumed to be related to the defense response to other organisms (GO:0098542) (Supplementary Table S10). The functional annotation data in Supplementary Table S10 showed that among these sequences related to the defense response to other organisms, 2800–2900 sequences are predicted to be involved in the innate immune response (GO:0045087). Approximately 30–35% of these sequences are predicted to be involved in plant hypersensitive response (GO:0009626) and 14–15% in the pattern recognition receptor signaling pathway (GO:000221) (Supplementary Table S11). Among the sequences related to the pattern recognition receptor signaling pathway (GO:000221), 23–30% may be involved in the pathogen-associated molecular pattern (PAMP) receptor signaling pathway (GO:0002752). Approximately 11–15% of the sequences are predicted to be involved in the cell surface pattern recognition receptor signaling pathway (GO:0140426) (Supplementary Table S11).

## 3. Discussion

Citrus is a very important fruit crop in many parts of the world, valued for its nutritional properties. The HLB disease that is now prevalent in many citrus-growing regions of the U.S.A. has caused reduced citrus acreage and enormous financial damage because of the incurable nature of this disease. In the state of Florida, a major producer of citrus in the U.S.A., HLB has decreased citrus production by 74% since the disease arrived in 2005 [20]. The value of the citrus crop declined by 29% in 2021–22 compared to the previous year [21]. Disease-resistant varieties would protect the industry from such losses and provide a sustainable solution for citrus cultivation. Australian limes are valuable as sources of tolerance/resistance to HLB. The high-quality phased genomes reported here will be useful for analyzing the breeding populations and identifying the candidate genes/quantitative trait loci (QTLs) associated with an HLB-resistant phenotype. We generated over 4000 F1 (first-generation) and advanced (second-generation) hybrids with Australian lime parentage [7,8]. Many novel hybrids exhibit HLB tolerance/resistance traits. The analysis of these hybrids using the genomic information developed in the present study would facilitate the identification of resistance loci and the pre-selection of putatively valuable hybrids from the breeding populations.

### 3.1. Characterization of the Three Australian Lime Genomes

For the three Australian limes, the size of the assembled haploid nuclear genomes was 337 Mb for *C. australasica*, 303 Mb for *C. inodora*, and 378 Mb for *C. glauca*. The nine chromosome-scale scaffolds represent 86.1–91.4% of the total genome in de novo assemblies with N50 value of 29–39 Mb. Recently, the genome of *C. australis* has been reported [19]. Based on the genome size, *Citrus glauca* was the largest (primary and alternate haplotype: 376 Mb and 379 Mb, respectively) among all known Australian lime genomes (Supplementary Table S12). The sizes of lemon genomes reported are *C. limon* cv. Eureka v1.0 (316 Mb), *C. limon* L. Burm f. v1.0 (312 Mb), and *C. limon* cv. Xiangshui genome v1.0 (365 Mb) [22,23,24]. The haploid genome of *C. medica* (citron) is the largest known citrus genome at 406 Mb [25]. The haploid genome assembly of Majia pummelo (*C. maxima* v1.0 Cupi Majiayou) was reported to be 368 Mb [26], smaller than *C. glauca*. The genome sizes of *C. australasica* and *C. inodora* are similar to the citrus varieties like sweet orange (*C. sinensis*), mandarin (*C. reticulata*), and lemon (*C. limon*) [23,27,28]. The *C. australasica* genome is 7 Mb larger than the haplotype genome of another Australian lime, *C. australis*, estimated to be 331 Mb [19]. The genome size of trifoliate orange (*C. trifoliata*), a close deciduous relative of evergreen citrus, was found to be the smallest at 265 Mb. The size of *C. inodora* genome is 303 Mb, comparable to *C. clementina* (301.4 Mb) [29].

### 3.2. Significant Size Variations between Primary and Alternate Haplotypes of Australian Limes

Improvements in genome sequencing approaches, including long-read technology and the development of assembly tools, have made it possible to achieve efficient and accurate chromosome-level reference genomes for citrus [30,31]. Recently, haplotype-resolved genomes of citrus accessions have been reported for *C. limon* L. Burm f. [23], sweet orange (an introgressive hybridization of pummelo and mandarin) [32], and a wild Australian lime citrus species, *C. australis* [19]. Using integrated PacBio long-read and Hi-C (chromatin cross-linked) methodologies, we constructed haplotype-level genomes of the three novel Australian limes. Genome and synteny analysis between two haplotypes of each of the three Australian limes showed significant conserved regions (83–93%) between them (Figure 1 and Figure 2). Haplotype-resolved genome assemblies in plants often have substantial differences among haplotypes in chromosomal rearrangements, sequence insertions, and expressions of specific alleles that contribute to the acquisition of the biological characteristics of plant species [33,34]. In the present study, significant differences were observed in chromosome lengths and the synteny between Australian lime genomes and reference genomes of *C. clementina* and *C. trifoliata* (Figure 3). Some of these differences may be due to assembly errors in the genomes. They may also indicate evolutionary divergence, genetic adaptation, or genomic rearrangements specific to Australian limes.

### 3.3. Australian Lime Gene Count Is Similar to Other Known Citrus Species

Annotated genomes for wild citrus species offer an opportunity to identify the genes driving HLB resistance and their relation to genes in commercial citrus varieties. All three genomes were annotated using a combination of de novo gene calling and identification of known protein models. The number of genes in the wild Australian lime genomes were comparable to those of cultivated citrus.

### 3.4. R Genes Scattered across Nine Chromosomes in Australian Limes

The resistance genes (*R* genes) present in plant genomes contribute to resistance or tolerance to various pathogens [35]. Elevated levels of tolerance/resistance against HLB observed in Australian limes may be associated with specific *R* genes. Well-recognized *R* genes consist of a nucleotide-binding domain (NB-ARC) and a leucine-rich repeat (LRR) domain, known as NLRs; these can be subdivided into TIR–NBS–LRR (TNL) or CC–NBS–LRR (CNL) types based on the structure of N-terminal domains. In this study, the identified NLRs ranged from 319 to 651 in the five citrus species, with the two commercial cultivars having slightly higher NLRs than the Australian limes (Table 5). Approximately half of the total NLRs within each species fell in the CNL or TNL groups, with the number of CNLs similar to TNLs in *C. glauca* but 30–90% more in other species.

The distribution of *R* genes was uneven in the genome, with most *R* genes located on chromosomes 1, 3, 5, and 7 (Figure 4 and Supplementary Figure S4). Many *R* genes of similar categories formed large clusters, likely due to gene duplication during evolution. Identifying the functional motif sequences unique to Australian limes may shed light on the HLB tolerance/resistance mechanism. The knowledge generated may provide a resource for developing tools for marker-assisted breeding in hybrids with Australian lime parentage.

### 3.5. PP2 and CalS—Proposed Roles in HLB

The Phloem-limited intracellular Gram-negative bacterium CLas is associated with HLB disease. Phloem cells in the HLB-infected citrus undergo structural modifications, including cell wall thickening, Callose and Phloem protein induction, and cellular plugging. The roles of *CalS* and *PP2* were well studied in sweet oranges infected with HLB; sieve elements were reported to be blocked by filamentous protein material containing PP2 [36]. The gene expression of *PP2* was also shown to be upregulated in the leaves of HLB-infected sweet orange plants compared to healthy plants [37]. In addition, PP2 transcripts were reported to be upregulated in an HLB-susceptible citrus variety compared to those of a tolerant variety [38]. Recent studies have suggested that *CalS* and *PP2* genes were upregulated in the shoots but downregulated in root tissues [36]. These studies indicate that *CalS* and *PP2* expression and Phloem plugging may play a crucial role in the onset of disease symptoms in susceptible citrus. Australian lime genomes are predicted to have more *PP2* and *CalS* genes than other citrus accessions. This may be related to higher HLB tolerance/resistance levels reported in Australian limes; however, experimental evidence to validate the roles of *PP2* and *CalS* genes in HLB disease development is needed. High-throughput single-cell transcriptome (scRNA-seq) sequencing may be useful for understanding the disease progression associated with CLas movement [39].

### 3.6. De Novo Transcriptome Analysis Unveiled Genes Associated with “Response to Other Organisms”

The percentages of host transcript recoveries of 81–94% (Table 7) are in agreement with our previous study [40], especially considering that the plant samples were obtained from field sources and are likely to have other pests and pathogens. TransDecoder predicted that 73–76% of de novo transcriptome contigs of the three genomes harbor ORFs. Among these contigs, 62–66% were functionally annotated. The annotation data at Level 3 of the GO category of Biological Process showed the presence of genes that are involved in “Response to Other Organisms (GO:0009725)” (Supplementary Table S11). Approximately one-third of the genes related to the defense response to other organisms are predicted to be involved in the innate immune response that can lead to pattern-triggered immunity (PTI) and effector-triggered immunity (ETI), as well as genes responsible for hypersensitive response (HR) in all three genomes. The functional relevance of the genes involved in PTI/ETI needs to be further investigated to understand the genetic mechanism of the HLB resistance phenomenon in Australian limes. The trees analyzed for gene expression did not have HLB but may carry other citrus pathogens.

## 4. Materials and Methods

### 4.1. Plant Materials and Sequencing

Three individual trees of *Citrus australasica* (IVNO 6502), *C. inodora* (IVNO 2781), and *C. glauca* (IVNO 6206) were sampled for sequencing the genome (Supplementary Table S1). The Givaudan Citrus Variety Collection (GCVC; https://citrusvariety.ucr.edu; accessed on 20 January 2021), University of California Riverside, California, maintains the trees. Young tender leaf tissue was collected and immediately placed in liquid nitrogen for subsequent high-molecular-weight DNA extractions from approximately 40 g of leaf material [41,42]. The genomic DNA samples were sheared with a gTube to an average fragment length of 13 kb, and libraries were constructed with the SMRTBell Express Template Prep kit 2.0 (Pacific Biosciences, Menlo Park, CA, USA). The libraries were sequenced on one 8M-SMRTcell for each sample in the Sequel II system using circular consensus long-read sequencing (CCS) (NRGene, Inc., San Diego, CA, USA).

### 4.2. Genome Assembly and Chromosome-Scale Scaffold

The raw FASTQ files for each genome were processed to construct de novo genome assemblies using Hifiasm [43] using default parameters. Hifiasm assembly statistics were viewed using Bandage, v 0.8.1 [44], and a Python script (get_asm_stats.py) from PacBio Assembly Tool Suite (PacBio, Menlo Park, CA, USA). Chromosome-scale scaffolds for each genome were generated using a Proximo Hi-C 4.0 Kit Hi-C protocol, following the manufacturer’s instructions [45]. Briefly, intact cells from each sample were cross-linked using a formaldehyde solution, digested using the DPNII restriction enzyme, and proximity ligation was performed with biotinylated nucleotides to create chimeric molecules composed of fragments from different regions of the genome (Phase Genomics, Seattle, WA, USA). The chimeric molecules were captured with streptavidin beads, and sequencing was performed on the Illumina NovaSeq platform (Supplementary Table S13). Reads were aligned with their respective draft assemblies using BWA-MEM [46], and PCR duplicates were flagged using SAMBLASTER [47]. Alignments were then filtered using samtools [48], and FALCON-Phase was used to correct likely phase-switching errors in the primary and alternate haplotigs [49]. Phase Genomics’ Proximo Hi-C genome scaffolding platform was used to create chromosome-scale scaffolds from FALCON-Phase’s phase assembly. Juicebox was then used to correct scaffolding errors [50]. The metadata generated by FALCON-Phase scaffold phasing were used to produce a diploid, fully phased, chromosome-scale set of scaffolds. The final chromosome-scale scaffolds for each genome were then ordered and oriented to correspond with the *Citrus clementina* (v1.0) genome from Citrus Genome Database (CGD).

### 4.3. BUSCO Score Analysis

A quantitative assessment of genome assembly and the predicted protein sequences was performed using BUSCO v.5.4.4 [51]. The two lineages used were eudicots_odb10 and viridiplantae_odb10 [17].

### 4.4. Synteny Analysis between Primary and Alternate Haplotypes

The syntenic relationship between primary and alternate haplotypes of the Australian lime genomes was visualized using minimap2 [52]. The output file (.paf) was used to generate the link file using an in-house Python 3.12. script (create_rideogram_paf_linkage.py). The final plots were constructed using the Pafr and ggplots packages in open-source Rstudio [53].

### 4.5. Genomic Variation of Australian Limes with Reference Genomes

To identify the genomic variation of Australian limes compared with *C. clementina* [29] and *C. trifoliata* [54], SyRI was used with default parameters [55]. The primary and alternate haplotype sequences were used as queries against the two reference genomes. Assemblies were mapped using minimap2, and .sam files were generated. Samtools were used to convert .sam files to BAM, followed by indexing and sorting bam files [48]. SyRI was used to read the .bam files, and .vcf files containing variants, including single-nucleotide polymorphisms (SNPs), inversions, translocations, duplications, insertions, deletions, and highly diverged regions, were generated. Data were visualized using the Pafr and ggplot2 libraries in RStudio [53]. Approximate per-base differences (“divergence”) between the primary and alternate haplotypes, alignment length, and sequence divergence were predicted using minimap2.

### 4.6. Transcriptome Analysis

A transcriptome analysis of the Australian lime genomes was performed using Illumina HiSeq-NovaSeq (150 PE) (Illumina, San Diego, CA, USA). RNA was extracted from various tissues of *C. australasica*, *C. inodora*, and *C. glauca* trees collected from GCVC, Riverside, CA. Total RNA was prepared from multiple tissues (Table 7). RNA quality and integrity were evaluated using an Agilent 2100 Bioanalyzer (Agilent Technologies, Santa Clara, CA, USA). A total of 20 barcoded cDNA libraries were prepared and sequenced (Genewiz, Inc., South Plainfield, NJ, USA). Raw reads with a Phred score (Q) of 30 were selected for analysis after removing adapter sequences. Quality control was performed using the FastQC toolkit (https://www.bioinformatics.babraham.ac.uk/projects/fastqc/; accessed on 8 August 2021).

A quality-filtered RNA-seq read of each sample was mapped to the corresponding genomes using CLC genomics workbench v 24.0.1 (Qiagen, San Diego, CA, USA), from which samples with >68% read mapping results (Table 7) were processed for transcript discovery using a transcript discovery tool in CLC genomics workbench. First, a pool of the quality-filtered RNA-seq reads of selected samples in each of the three Australian limes was used for “Large Gap Read Mapping” in CLC genomics workbench using their corresponding genome with the structural annotation data (gene and transcript) as a reference.

### 4.7. Gene Annotation of the Assemblies

We identified repetitive elements in both the primary and alternative assemblies using RepeatModeler version 2.0.3 and soft-masked them with RepeatMasker version 4.1.2 [56]. The results of RepeatModeler were merged with RepeatMasker’s internal repeat database to mark unaccounted repeats, and all repeats associated with rRNA sequences were ignored. Gene annotation was accomplished with two rounds of BRAKER v2.1.6 analysis per assembly [57]. The first round was based on RNA evidence obtained from aligning *C. australasica* RNA sequencing data and citrus hybrid sequencing data using STAR [58]. The second round was based on protein evidence collected from known citrus species using GeneMark-EP’s ProtHint pipeline [59]. We merged the results of both BRAKER runs with TSEBRA, creating a combined annotation for both assemblies [60]. The merged annotation file was filtered by structure and function using gFACs version 1.1.2 and EnTAP version 0.10.8 [61,62].

Genes specific to Australian lime species were identified by running Orthofinder, version 2.5.4. [63]. All primary protein gene models predicted for the primary assemblies were used. The protein gene models for six other citrus species—*C. australis* [19], *C. clementina* [29], *C. limon* [23], *C. maxima* [26], *C. sinensis* [27], and *C. trifoliata* [54]—were also obtained. These protein files were filtered to include only primary sequences when necessary, preventing isoforms from being labeled as orthologs. Orthofinder was run using MAFFT version 7.508 to obtain multiple sequence alignment (MSA) files [64]. Orthogroups containing only one or more genes from all four Australian lime species, consisting of *C. australasica*, *C. australis*, *C. glauca*, and *C. inodora*, were identified. We also confirmed the number of unplaced genes and genes in single-species orthogroups. We performed GO term enrichment on these genes of interest by taking the gene annotation data obtained from EnTAP for the three taxa and processing the identified GO terms through AgriGO v2.0 [65].

### 4.8. Identification and Classification of R Genes and Chromosomal Localization

In addition to the Australian species, we included two commercial citrus species: sweet orange (*C. sinensis*) and clementine mandarin (*C. clementina*). The latest versions of their whole-genome protein sequences were downloaded from the Citrus Pan-genome to Breeding Database (http://citrus.hzau.edu.cn/orange/; accessed on 16 June 2023). The genome sequences of each citrus species were screened against the Pfam database using CLC Genomics Workbench v 20.0 (Qiagen, Germantown, MD, USA) with an *E*-value < 0.001 for detecting the NBS (nucleotide-binding site) domain (PF 00931) characteristic of most *R* genes in plants. The identified *R* genes were then submitted to the online tool InterPro [66] for domain prediction with default parameters. The *R* genes from each species were mapped to the corresponding chromosomes using the “Gene Location Visualize” function in TBtools [67] based on their chromosomal coordinates obtained from the annotation files. The chromosomal locations of *R* genes in three Australian limes are shown in the Circos plot (Figure 4 and Supplementary Figure S4).

### 4.9. Identification of Phloem Protein 2 and Callose Gene Family

Phloem protein 2 (*PP2*; PF14299) [68] and Callose synthase (*CalS*; PF14288) [69] were identified using the hidden Markov model (HMM). Briefly, the HMM profiles using HMMER v3.4 (http://hmmer.org/; accessed on 25 October 2022) were mapped on protein sequences [70] from *C. australasica*, *C. inodora*, and *C. glauca*. All the significant hits with positive scores were selected and mapped on chromosome-scale scaffolds. Graphical representations were constructed using ggplot2 in RStudio [53]. Multiple sequence alignments of amino acid sequences belonging to *PP2* and *CalS* from *C. australasica* (Ca), *C.inodora* (Ci), *C. glauca* (Cg), *C. sinensis* (Cs), *C. clementina* (Cc), *C. medica* (Cm), and *C. trifoliata* (Pt) were performed using ClustalX (version 2.2) (http://www.ebi.ac.uk/; accessed on 12 December 2023) with default parameters and built-in Jalview software version 2.11 (http://www.jalview.org/; accessed on 12 December 2023). The phylogenetic trees were generated using ClustalX, which implemented the maximum-likelihood method. Bootstrap values were calculated at 1000 iterations. The final phylogenetic tree was edited using ITOL (http://itol.embl.de/; accessed on 12 December 2023), as described earlier [71].

## 5. Conclusions

High-quality phased genomes of the three Australian lime genotypes with HLB resistance/tolerance are presented in this study. These trees were utilized as parents in our breeding program to generate novel citrus hybrids with Australian lime parentage. Many hybrids have been shown to exhibit HLB tolerance in greenhouse and field studies. The genomic resources developed here would help identify the QTLs for HLB resistance and facilitate the development of rapid screening methods for breeding populations. Because of the devastation of the citrus industry in the United States due to HLB, the novel hybrids emanating from the program will provide much-needed long-term solutions. The genome sequences generated will add to the repertoire of genetic and genomic resources that can be utilized to improve citrus cultivation.

## Figures and Tables

**Table 1:** Statistics of phased genome (primary and alternate haplotypes) assembly of the three Australian limes.

	*Citrus australasica*		*C. inodora*		*C. glauca*	
Haplotype	Primary	Alternate	Primary	Alternate	Primary	Alternate
Estimated genome size (Mb)	336.7	335.2	303.7	298.8	376.4	379.2
Number of scaffolds	270	270	299	299	83	83
N50 (Mb)	30.0	30.2	28.9	28.9	36.9	36.7
N90 (Mb)	23.7	22.3	26.9	25.7	30.9	30.2
Largest scaffold (Mb)	47	46.7	41.6	41.8	57.9	58.9
Largest scaffold (%)	13.9	13.9	13.7	14	15.4	15.5
9 Chromosomes (Mb)	290	289.2	276.9	272	344.3	345.1
9 Chromosomes (% of genome)	86.1	86.2	91.2	91	91.5	91
% GC content	36.5%	36.4%	36.4%	36.4%	37.1%	37.1%
NCBI accession	PRJNA924094	PRJNA924095	PRJNA924048	PRJNA924114	PRJNA924119	PRJNA924121

**Table 2:** BUSCO analysis of the three genomes. We used BUSCO v.5.4.4 with two lineages: eudicots_odb10 and viridiplantae_odb10 [17].

		Genome ^a^		Annotation ^b^	
Species	Haplotype	Eudicot (%)	Viridiplantae (%)	Eudicot (%)	Viridiplantae (%)
*Citrus australasica*	Primary	98.70	99.10	96.80	99.10
	Alternate	98.80	99.20	97.50	99.10
*C. inodora*	Primary	96.68	96.70	93.30	94.20
	Alternate	96.68	96.70	92.80	94.60
*C. glauca*	Primary	96.90	97.20	94.50	97.40
	Alternate	96.90	97.20	94.90	96.70

^a^ based on the genome assembly; ^b^ based on the annotation of predicted proteins from the genome assembly.

**Table 3:** Genomic variation between the primary and alternate haplotypes in the three Australian limes. Haplotypes were mapped with minimap2, and variant call format (vcf) files were generated using Synteny and Rearrangement Identifier (SyRI).

Species	SNP ^a^	Inversion	Translocation	Duplication	Insertion	Deletion	HDR ^b^
*Citrus australasica*	1,753,794	120,866	148,742	6251	133,808	134,289	8448
*C. inodora*	2,245,738	185,236	158,654	12,899	167,345	147,099	11,897
*C. glauca*	2,216,981	211,143	152,934	10,614	158,513	143,167	9386

^a^ single-nucleotide polymorphism; ^b^ highly diverged regions.

**Table 4:** Annotations of three Australian lime genomes.

Species	*Citrus australasica*		*C. inodora*		*C. glauca*	
Haplotype	Primary	Alternate	Primary	Alternate	Primary	Alternate
Genes (BRAKER)	27,358	25,461	28,176	27,665	30,067	33,673
Proteins (gFACs)	27,348	25,451	28,173	27,664	30,066	33,672
EnTAP-Functional Filtering	22,942	22,912	24,330	23,885	24,136	24,448
Exons	139,684	137,485	143,260	141,940	157,064	161,029
Introns	109,469	109,259	111,706	110,794	123,639	123,804
CDS	139,709	137,511	143,337	142,039	157,079	161,049
Transcripts	30,242	28,257	31,632	31,245	33,440	37,246

**Table 5:** Number of *R* genes in citrus species. NLR, *R* genes with nucleotide-binding (NB) and leucine-rich repeat (LRR) domains; CNL, NLRs with coiled-coil (CC) N-terminal domain; TNL, NLRs with Toll/Interleukin-1 receptor/resistance protein (TIR) N-terminal domain; NL, NLRs with N-terminal domains different from CNL or TNL.

Citrus Species	NLR	CNL	TNL	NL
*C. australasica* ^a^	576	195	119	262
*C. australasica* ^b^	545	172	129	244
*C. inodora* ^a^	319	109	69	141
*C. inodora* ^b^	325	107	71	147
*C. glauca* ^a^	449	114	105	230
*C. glauca* ^b^	459	116	112	231
*C. clementina*	579	205	104	270
*C. sinensis*	651	214	145	292

^a^ primary haplotype; ^b^ alternate haplotype.

**Table 6:** Identified *Phloem protein 2* (*PP2*) and *Callose synthase* (*CalS*) in *C. australasica*, *C. inodora*, and *C. glauca*. Pfam protein profiles of PP2 (PF14299) and Callose synthase (PF14288) from Pfam v35.0 was used for the analysis using HMM v3.5.

Species	Haplotype	Phloem Protein (PP2)	Callose Synthases (CalS)
*Citrus australasica* v1.0	Primary	17	11
	Alternate	30	11
*C. inodora* v1.0	Primary	25	9
	Alternate	26	11
*C. glauca* v1.0	Primary	19	9
	Alternate	37	10
*C. clementina* v1.0		41	15
*C. sinensis* v2.0		34 (37 ^a^)	11 (17 ^a^)
*C. medica* v1.0		34 (36 ^a^)	10 (18 ^a^)
*C. trifoliata* v1.3		25 (39 ^a^)	8 (11 ^a^)

^a^ protein isoforms.

**Table 7:** The summary of “Large Gap Read Mapping” results of a pool of quality-filtered RNA-seq reads obtained from various tissue samples of *C. australasica*, *C. inodora*, and *C. glauca* against their primary and alternative haplotype genomes.

	*C. australasica*		*C.inodora*		*C. glauca*	
Read Mapping Results	No. of Reads in Millions	Percentage	No. of Reads in Millions	Percentage	No. of Reads in Millions	Percentage
Total reads	585.6		415.1		470.1	
Mapped reads	472.6	80.7	385.9	93	443.1	94.3
Reads in pairs (PE)	414.7	70.8	361.1	87	409.6	87.1
Broken PE	57.9	9.9	24.7	5.95	33.6	7.1
Not mapped	112.9	19.3	29.2	7	26.9	5.7

## Data Availability

Raw sequences’ (genome assembly, DNA-seq, and RNA-seq) data generated in this study have been deposited in NCBI Sequence Read Archive (SRA) for *C. australasica*: Biosample SAMN32745830 and Bioproject: PRJNA924094 (primary haplotype) and PRJNA924095 (alternate haplotype); *C. inodora*: Biosample: SAMN32744904 and Bioproject: PRJNA924048 (primary haplotype) and PRJNA924114 (alternate haplotype); *C. glauca*: Biosample: SAMN32746385 and Bioproject: PRJNA924119 (primary haplotype) and PRJNA924121 (alternate haplotype).

## Supplementary Materials

The following supporting information can be downloaded at: https://www.mdpi.com/article/10.3390/plants13111460/s1, Figure S1: FALCON-Phase assembly used to create chromosome-scale scaffolds using Hi-C proximo method; Figure S2: Mapping of Australian lime genomes on reference genomes *Citrus clementina* and *C. trifoliata*; Figure S3: Per-base divergence of nine chromosome-scale scaffolds of *Citrus australasica*, *C. inodora*, and *C. glauca*; Figure S4: Characterization of the three Australian lime alternate haplotype genomes of (A) *C. australasica*, (B) *C. inodora*, and (C) *C. glauca*; Figure S5: Gene Ontology (GO) classification of annotated sequences of *C. australasica*; Figure S6: Gene Ontology (GO) classification of annotated sequences of *C. inodora*; Figure S7: Gene Ontology (GO) classification of annotated sequences of *C. glauca*; Figure S8: Domain architecture of *Serine/threonine-protein kinase* of *C. australasica*, *C. inodora*, and *C. glauca*; Table S1: Details of accessions sequenced and PacBio data statistics of *C. australasica*, *C. inodora*, and *C. glauca*; Table S2: Chromosome-scale scaffolds of three Australian limes; Table S3: Genomic variation between the primary and alternate haplotypes in three Australian limes; Table S4: Orthogroup statistics of nine citrus species, including four Australian limes; Table S5: List of samples used for transcriptome analysis and read mapping yields; Table S6: Summary of de novo transcriptome assembly of quality-filtered paired-end RNA-seq reads of three Australian lime species; Table S7: Number of genes and transcripts predicted in each chromosome of the primary and alternate haplotypes; Table S8: Completeness assessment of de novo assemblies of three Australian lime species; Table S9: Summary of ORF prediction, followed by GO annotation of de novo assembly of three Australian lime species; Table S10: Number of annotated sequences in the de novo assemblies of three Australian lime species; Table S11: Functional annotation of a subset of genes that are involved in innate immune response (GO:0045087); Table S12: Genome size comparison of cultivated citrus species and the Australian limes; Table S13: Summary of Hi-C analysis for generating chromosome-scale scaffolds of *Citrus australasica*, *C. inodora*, and *C. glauca*.
