# Supplementary material for: Chromosome-Scale, De Novo, Phased Genome Assemblies of Three Australian Limes: Citrus australasica, C. inodora, and C. glauca
Source: Plants (Basel). 2024 May 24;13(11):1460. doi: 10.3390/plants13111460 (PMC11174732; doi:10.3390/plants13111460)
Supplement: Supplementary file 1 [file plants-13-01460-s001.zip › plants-2947233-Supplementary materials.pdf]

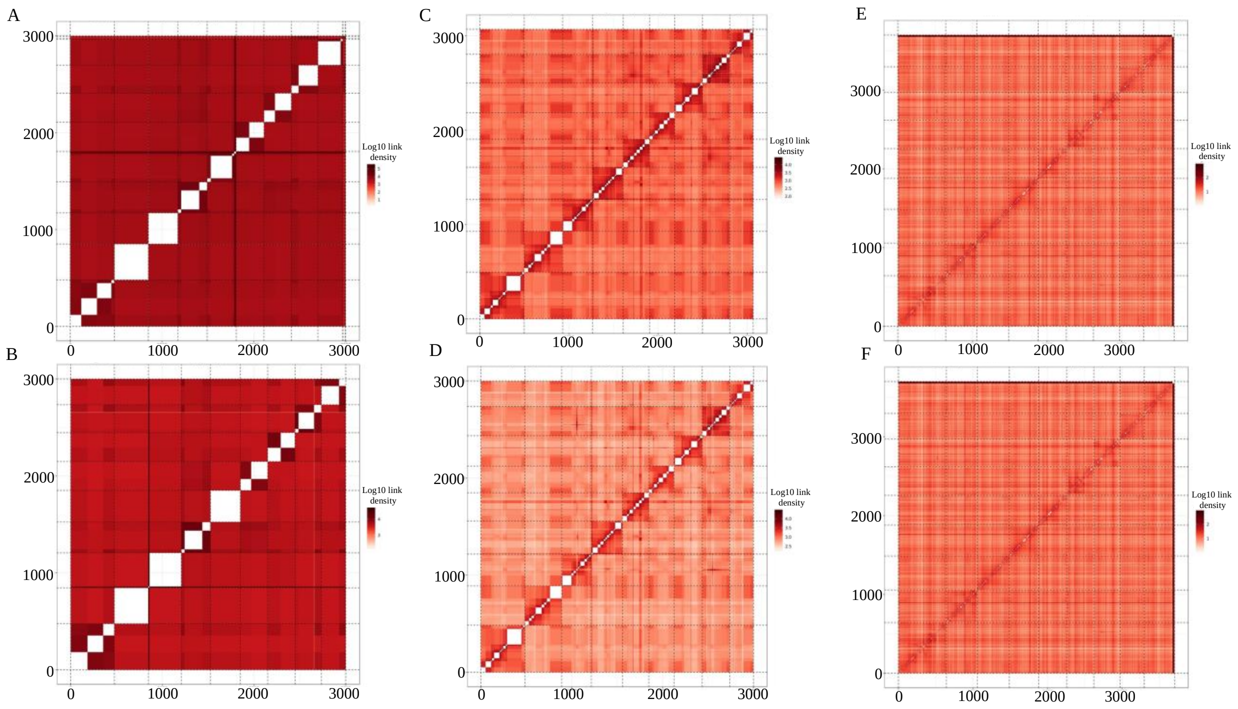


**Supplementary Figure S1** FALCON-Phase assembly was used to create chromosome-scale scaffolds using Hi-C proximo method. Juicebox was then used to correct scaffold errors. Nine large scaffolds are displayed for the three Australian lime genomes viz. A and B for *C. australasica*; C and D for *C. inodora* and E and F for *C. glauca*.

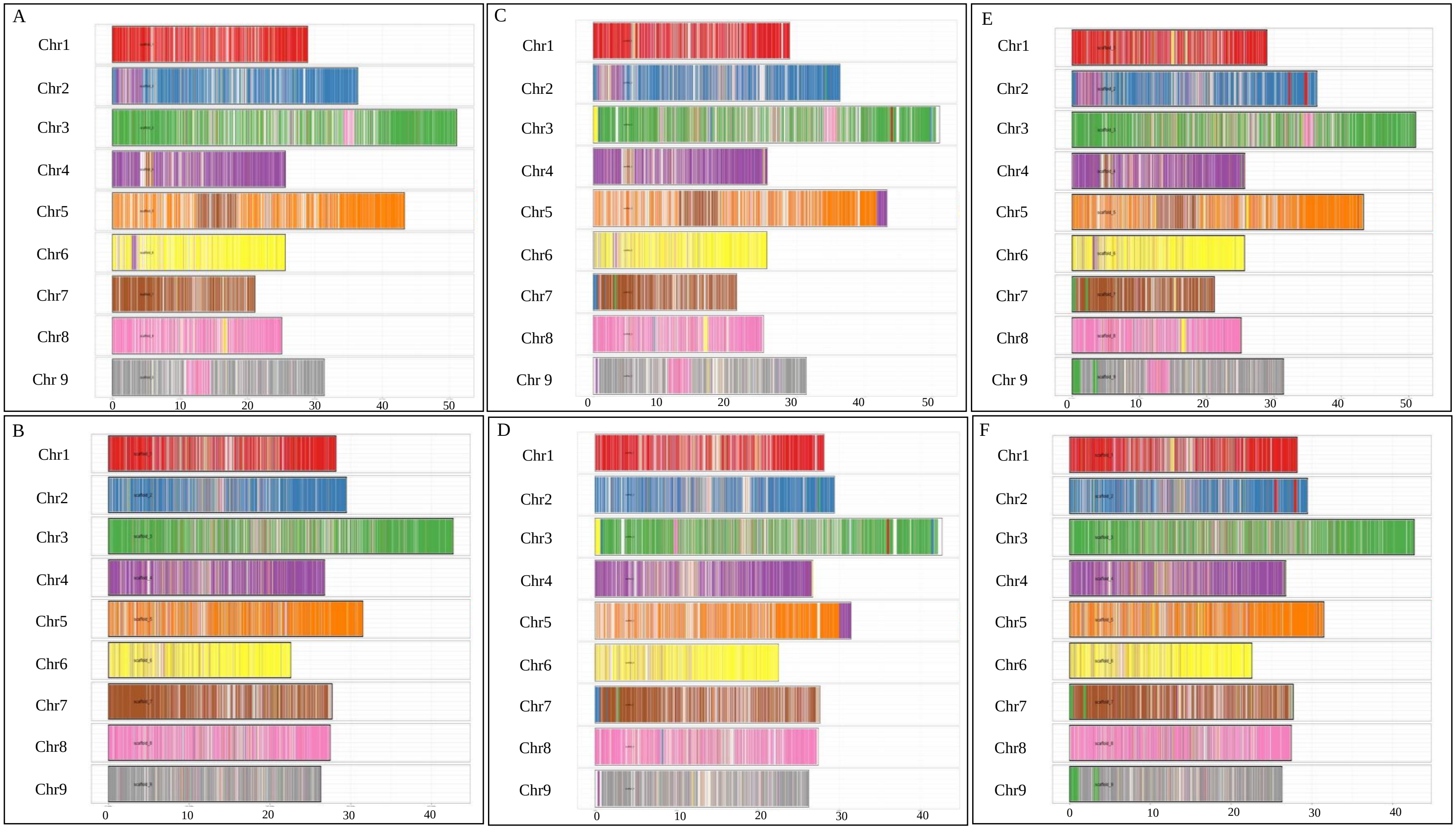


**Supplementary Figure S2** Mapping of Australian limes genomes on reference genomes *Citrus clementina* and *C. trifoliata*. Alternate haplotype sequences from A) *C. australasica*, C) *C. inodora,* and E) *C. glauca* were used as query sequences, while the target sequence (x-axis) represents the chromosomes of *C. clementina*. Alternate haplotype sequences from B) *C. australasica*, D) *C. inodora,* and F) *C. glauca* were used as query sequences, while the target sequence (x-axis) represents the

chromosomes of *C. trifoliata*. Chromosome scale positions are represented in Mb. Final plots were constructed using the Pafr and ggplots packages in open-source Rstudio [53].

A

| Chromosome # | *C. australasica* | *C. inodora* | *C. glauca* |
|---|---|---|---|
| Chr 1 | 0.05 | 0.05 | 0.04 |
| Chr 2 | 0.05 | 0.03 | 0.04 |
| Chr 3 | 0.06 | 0.04 | 0.05 |
| Chr 4 | 0.06 | 0.04 | 0.05 |
| Chr 5 | 0.05 | 0.05 | 0.05 |
| Chr 6 | 0.05 | 0.03 | 0.04 |
| Chr 7 | 0.05 | 0.04 | 0.04 |
| Chr 8 | 0.05 | 0.05 | 0.05 |
| Chr 9 | 0.06 | 0.04 | 0.05 |

B

| Species | *C. australasica* | | *C. inodora* | | *C. glauca* | |
|---|---|---|---|---|---|---|
| Haplotype | Primary | Alternate | Primary | Alternate | Primary | Alternate |
| Chr 1 | 0.08 | 0.08 | 0.07 | 0.07 | 0.09 | 0.09 |
| Chr 2 | 0.08 | 0.08 | 0.08 | 0.08 | 0.09 | 0.09 |
| Chr 3 | 0.08 | 0.08 | 0.08 | 0.07 | 0.09 | 0.09 |
| Chr 4 | 0.08 | 0.08 | 0.07 | 0.07 | 0.08 | 0.08 |
| Chr 5 | 0.09 | 0.08 | 0.08 | 0.08 | 0.09 | 0.09 |
| Chr 6 | 0.08 | 0.08 | 0.08 | 0.08 | 0.09 | 0.09 |
| Chr 7 | 0.08 | 0.08 | 0.07 | 0.07 | 0.09 | 0.09 |
| Chr 8 | 0.08 | 0.08 | 0.08 | 0.08 | 0.09 | 0.09 |
| Chr 9 | 0.08 | 0.08 | 0.08 | 0.08 | 0.10 | 0.10 |

C

| Species | *C. australasica* | | *C. inodora* | | *C. glauca* | |
|---|---|---|---|---|---|---|
| Haplotype | Primary | Alternate | Primary | Alternate | Primary | Alternate |
| Chr 1 | 0.09 | 0.09 | 0.09 | 0.09 | 0.10 | 0.10 |
| Chr 2 | 0.09 | 0.09 | 0.09 | 0.09 | 0.10 | 0.10 |
| Chr 3 | 0.09 | 0.09 | 0.09 | 0.09 | 0.10 | 0.10 |
| Chr 4 | 0.10 | 0.10 | 0.09 | 0.09 | 0.10 | 0.10 |
| Chr 5 | 0.09 | 0.09 | 0.09 | 0.09 | 0.10 | 0.10 |
| Chr 6 | 0.09 | 0.09 | 0.09 | 0.09 | 0.10 | 0.10 |
| Chr 7 | 0.09 | 0.09 | 0.09 | 0.09 | 0.10 | 0.10 |
| Chr 8 | 0.09 | 0.09 | 0.09 | 0.09 | 0.10 | 0.10 |
| Chr 9 | 0.09 | 0.09 | 0.09 | 0.09 | 0.11 | 0.11 |

**Supplementary Figure S3** Per-base divergence of nine chromosome-scale scaffolds of *C. australasica*, *C. inodora* and *C. glauca*. A) divergence between primary and alternate haplotype of three Australian limes B) divergence of Australian limes primary and alternate haplotypes with *C. clementina* C) divergence of Australian limes primary and alternate haplotypes with *C. trifoliata*. Approximate per-base differences ("divergence") were predicted using minimap2. Data were visualized using the Pafr and ggplot2 libraries in RStudio [53].

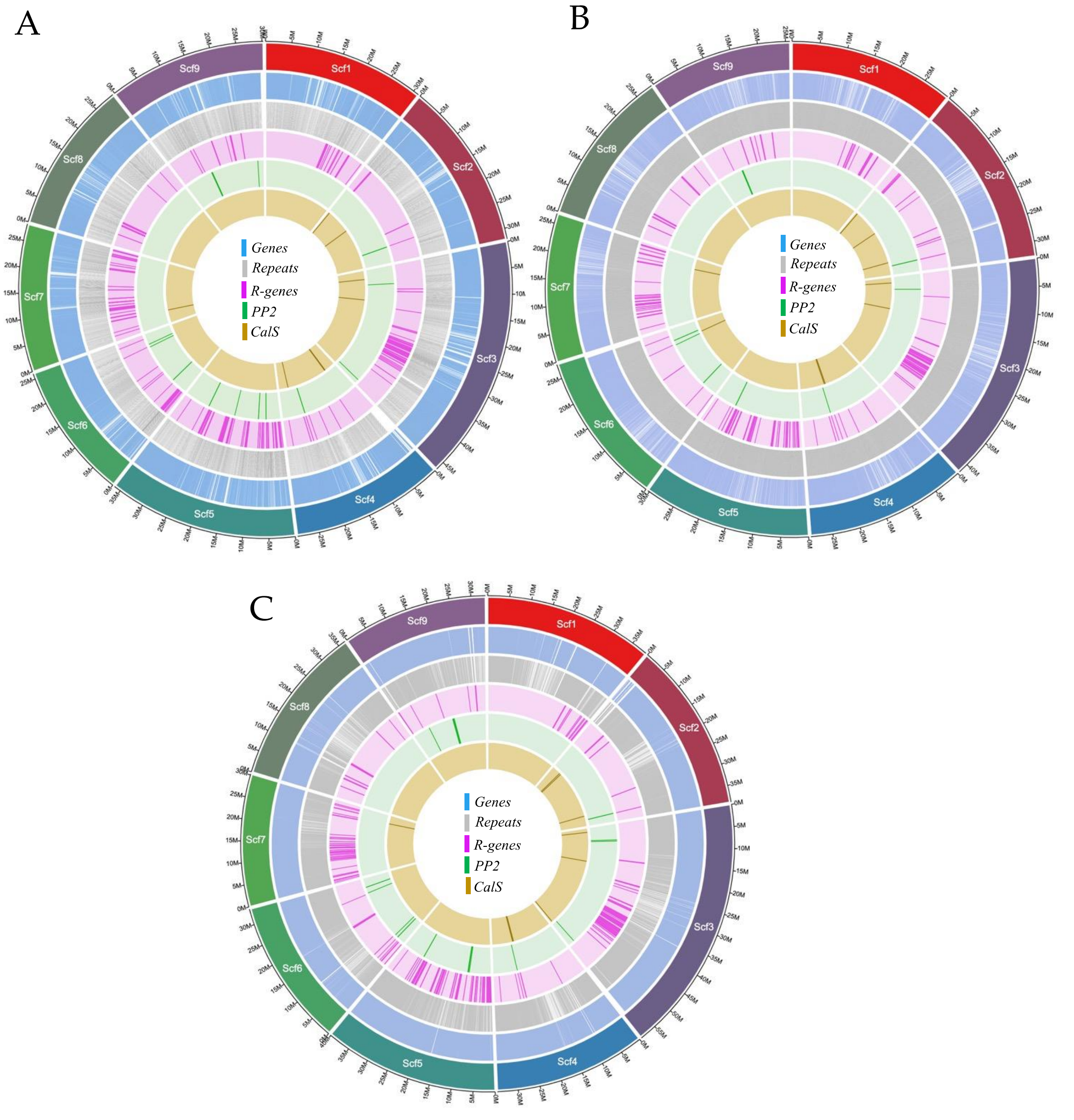
A
B
C
Scf1
Scf2
Scf3
Scf4
Scf5
Scf6
Scf7
Scf8
Scf9
Genes
Repeats
R-genes
PP2
CalS

**Supplementary Figure S4** Characterization of the three Australian lime alternate haplotype genomes of A) *C. australasica* B) *C. inodora* and C) *C. glauca*. From outer to inner rings: I. The nine assembled chromosome-scale scaffolds (in Mb) correspond to the nine chromosomes (Scf1− Scf9) of *Citrus clementina*. II. Locations of predicted gene models. III. Locations of predicted long terminal repeat (LTR) transposable elements (TEs). IV. Locations of the predicted nucleotide-binding site (NBS)-containing genes (R genes). V. Locations of predicted Phloem protein 2 (PP2) genes. VI. Locations of Callose synthase (*CalS*) genes. Circa was used to draw the circos plots.

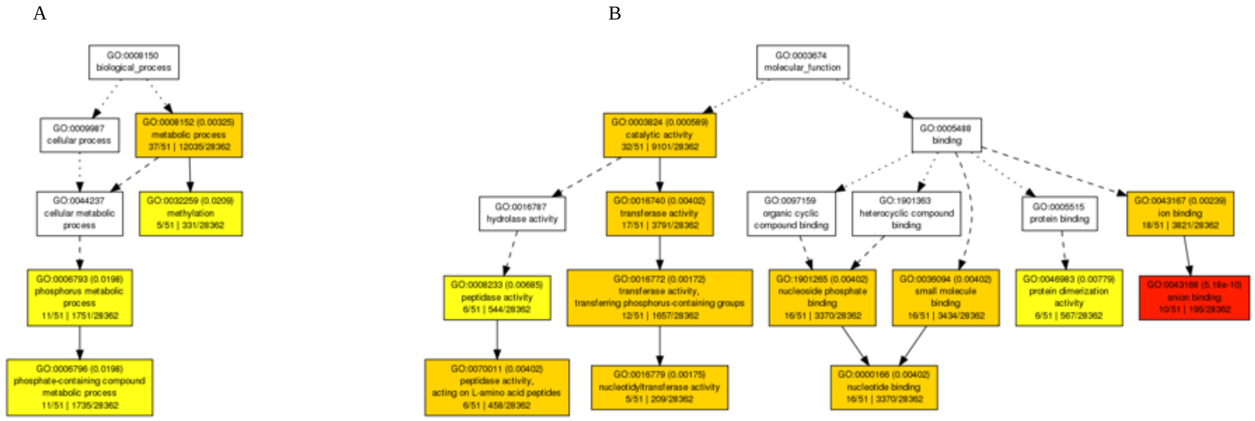


**Supplementary Figure S5** Gene Ontology (GO) classification of annotated sequences of *C. australasica* A) Biological Process, B) Molecular function

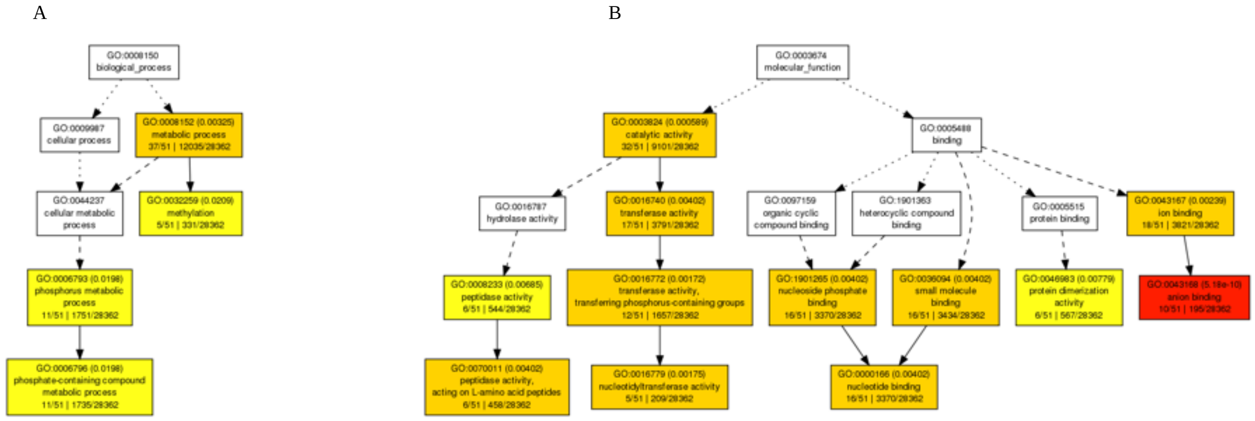


**Supplementary Figure S6** Gene Ontology (GO) classification of annotated sequences of *C. inodora* A) Biological Process, B) Molecular function

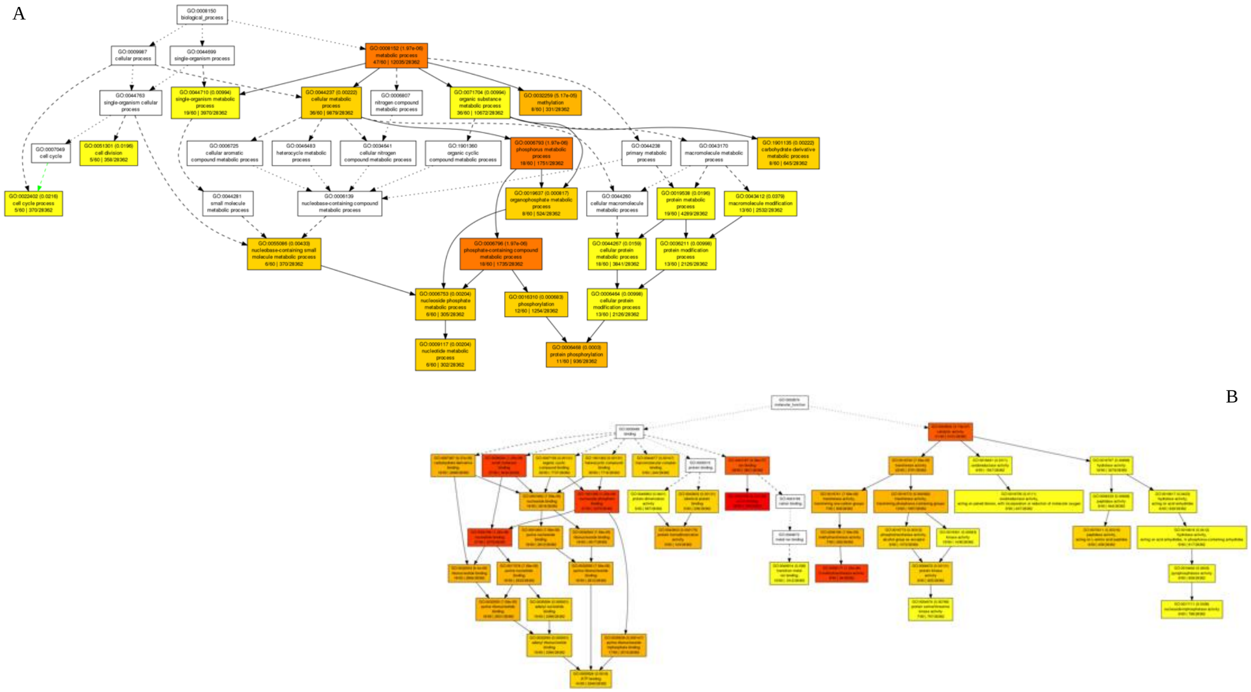
A
GO:0008150
biological_process
GO:0009987
cellular process
GO:0044699
single-organism process
GO:0008152 (1.97e-06)
metabolic process
47/60 | 12035/28362
GO:0044763
single-organism cellular process
GO:0044710 (0.00994)
single-organism metabolic process
19/60 | 3970/28362
GO:0044237 (0.00222)
cellular metabolic process
36/60 | 9879/28362
GO:0006807
nitrogen compound metabolic process
GO:0071704 (0.00994)
organic substance metabolic process
36/60 | 10672/28362
GO:0032259 (5.17e-05)
methylation
8/60 | 331/28362
GO:0007049
cell cycle
GO:0051301 (0.0196)
cell division
5/60 | 358/28362
GO:0006725
cellular aromatic compound metabolic process
GO:0046483
heterocycle metabolic process
GO:0034641
cellular nitrogen compound metabolic process
GO:1901360
organic cyclic compound metabolic process
GO:0006793 (1.97e-06)
phosphorus metabolic process
18/60 | 1751/28362
GO:0044238
primary metabolic process
GO:0043170
macromolecule metabolic process
GO:1901135 (0.00222)
carbohydrate derivative metabolic process
8/60 | 645/28362
GO:0022402 (0.0216)
cell cycle process
5/60 | 370/28362
GO:0044281
small molecule metabolic process
GO:0006139
nucleobase-containing compound metabolic process
GO:0019637 (0.000817)
organophosphate metabolic process
8/60 | 524/28362
GO:0044260
cellular macromolecule metabolic process
GO:0019538 (0.0196)
protein metabolic process
19/60 | 4289/28362
GO:0043412 (0.0379)
macromolecule modification
13/60 | 2532/28362
GO:0055086 (0.00433)
nucleobase-containing small molecule metabolic process
6/60 | 370/28362
GO:0006796 (1.97e-06)
phosphate-containing compound metabolic process
18/60 | 1735/28362
GO:0044267 (0.0159)
cellular protein metabolic process
18/60 | 3841/28362
GO:0036211 (0.00998)
protein modification process
13/60 | 2126/28362
GO:0006753 (0.00204)
nucleoside phosphate metabolic process
6/60 | 305/28362
GO:0016310 (0.000683)
phosphorylation
12/60 | 1254/28362
GO:0006464 (0.00998)
cellular protein modification process
13/60 | 2126/28362
GO:0009117 (0.00204)
nucleotide metabolic process
6/60 | 302/28362
GO:0006468 (0.0003)
protein phosphorylation
11/60 | 936/28362
B

C

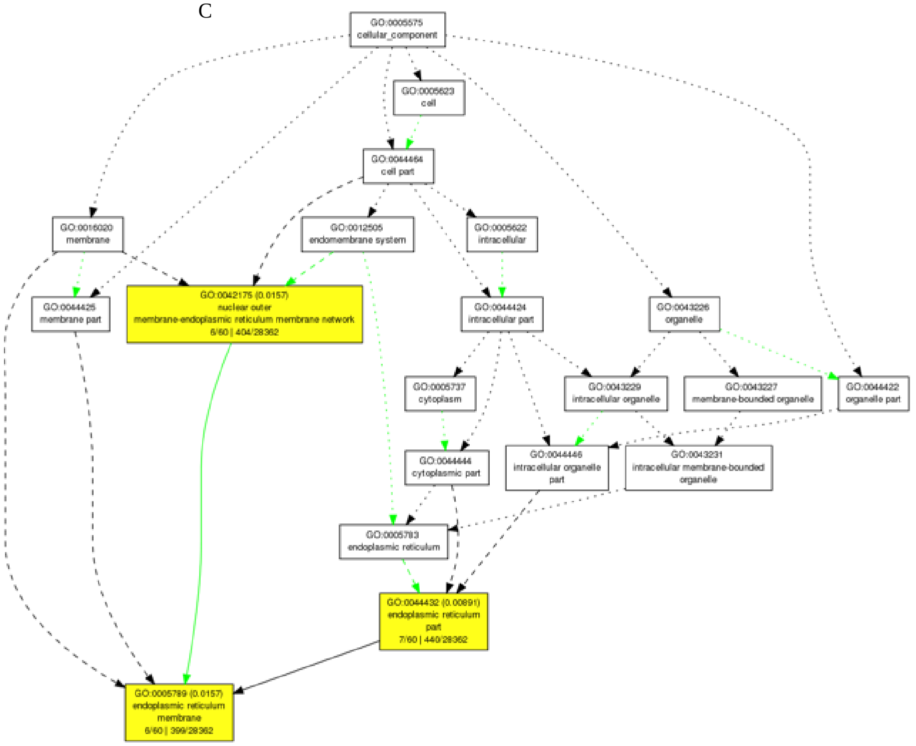


**Supplementary Figure S7** Gene Ontology (GO) classification of annotated sequences of *C. glauca* A) Biological Process, B) Molecular function, and C) cellular component

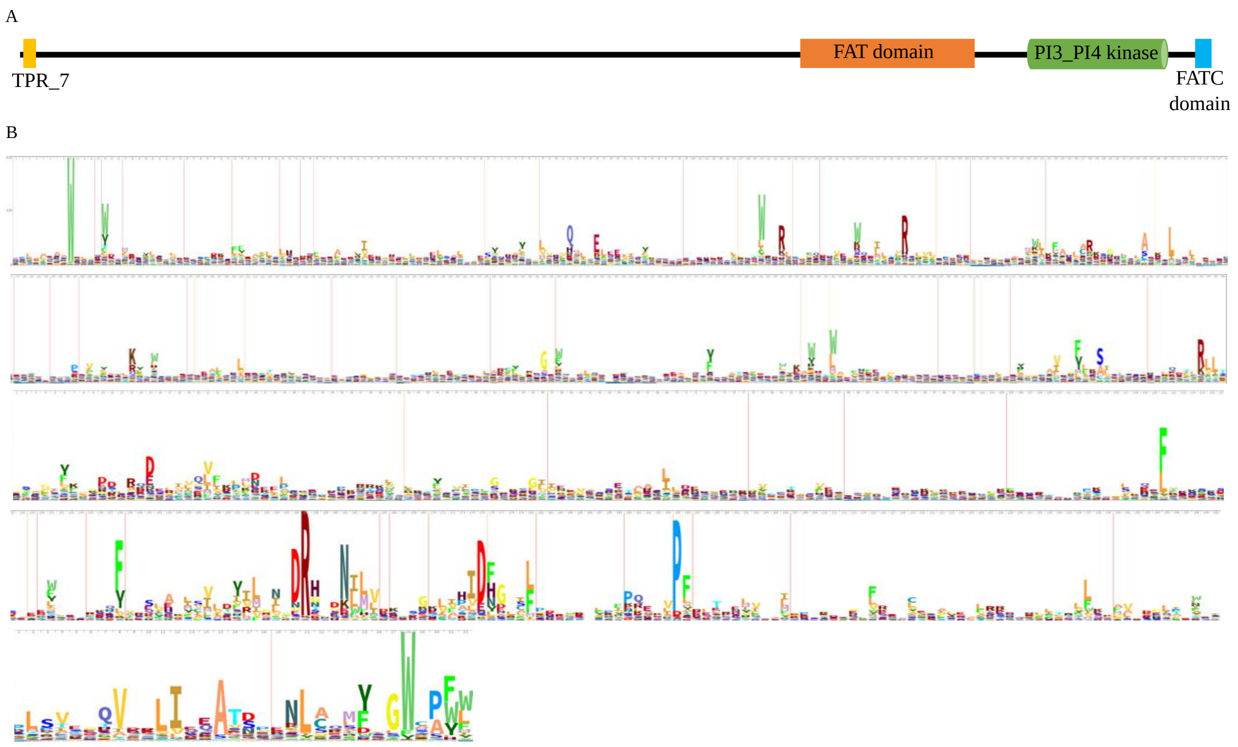


**Supplementary Figure S8** A) Domain architecture of Serine/threonine-protein kinase of *C. australasica*, *C. inodora*, and *C. glauca*. The protein consists of Phosphatidylinositol 3- and 4-kinase (*PI3_PI4 kinase*), FAT domain, FATC domain, and Tetratricopeptide repeat. Domain architecture was predicted using InterPro 95.0. B) represents the profile Hidden Markov Models (pHMMs) of Serine/threonine-protein kinase. Pfam v35.0 was used to create the logo.

## Supplementary Tables

**Supplementary Table S1.** Details of accessions sequenced and PacBio data statistics of *C. australasica*, *C. inodora,* and *C. glauca*. Sequencing was done using one SMRT Cell for each accession resulting in about 100X coverage of each genome.

| Species | IVNO [a] | CRC [b] | PI [c] | Minimum Read Length | Mean Read Length | Maximum Read Length | Data size (Gb) |
|---|---|---|---|---|---|---|---|
| *Citrus australasica* | 6502 | CRC 3672 | PI 312872 | 1,001 | 11,908 | 35,658 | 27.3 |
| *C. inodora* | 2781 | CRC 3784 | PI 539741 | 1,006 | 14,286 | 38,378 | 29.8 |
| *C. glauca* | 6206 | CRC 3463 | PI 539717 | 1,008 | 13,717 | 34,539 | 27.1 |

[a] IVNO identifies the individual tree in the field sampled for genome sequencing; [b] accession number of the Givaudan Citrus Variety Collection (CVC) of the University of California Riverside (UCR); [c] Plant Inventory accession number in the "Germplasm Resources Information Network" (GRIN) database of the United States Department of Agriculture (USDA).

**Supplementary Table S2.** Chromosome-scale scaffolds of three Australian limes. Chromosomes are arranged according to the reference genomes of *C. clementina* and *C. trifoliata*.

| | *C. australasica* | | *C. inodora* | | *C. glauca* | |
|---|---|---|---|---|---|---|
| Chromosome # | Primary | Alternate | Primary | Alternate | Primary | Alternate |
| 1 | 31.2 [a] | 31.1 | 30.3 | 29.9 | 39.0 | 38.7 |
| 2 | 31.6 | 32.1 | 33.3 | 32.6 | 39.0 | 38.8 |
| 3 | 47.0 | 46.7 | 41.7 | 41.8 | 58.0 | 58.9 |
| 4 | 29.9 | 29.2 | 28.9 | 28.9 | 34.5 | 34.7 |
| 5 | 36.4 | 36.3 | 30.2 | 30.3 | 39.7 | 40.1 |
| 6 | 26.5 | 26.0 | 26.9 | 27.6 | 33.1 | 33.6 |
| 7 | 28.4 | 28.2 | 28.8 | 26.8 | 31.0 | 30.2 |
| 8 | 29.1 | 29.2 | 28.2 | 28.3 | 37.0 | 36.7 |
| 9 | 29.7 | 30.2 | 28.6 | 25.8 | 33.1 | 33.3 |

[a] Chromosome sizes are shown (Mb).

**Supplementary Table S3.** Genomic variation between primary and alternate haplotypes in three Australian limes.

| *C. australasica* | | | | | | | |
|---|---|---|---|---|---|---|---|
| Chromosome# | SNP[a] | Inversion | Translocation | Duplication | Insertion | Deletion | HDR[b] |
| 1 | 168332 | 10610 | 9444 | 571 | 12971 | 13207 | 798 |
| 2 | 189239 | 8755 | 11821 | 606 | 15895 | 15720 | 940 |
| 3 | 283577 | 13914 | 26382 | 979 | 22394 | 22389 | 1418 |
| 4 | 193883 | 21755 | 13705 | 678 | 14286 | 14356 | 823 |
| 5 | 225085 | 15204 | 32059 | 1189 | 16941 | 17072 | 1121 |
| 6 | 159144 | 10239 | 10528 | 389 | 12094 | 12069 | 679 |
| 7 | 182272 | 8880 | 16308 | 524 | 14006 | 14049 | 951 |
| 8 | 174363 | 16004 | 13302 | 559 | 12714 | 12780 | 864 |
| 9 | 177899 | 15505 | 15193 | 756 | 12507 | 12647 | 854 |
| *C. inodora* | | | | | | | |
| Chromosome# | SNP[a] | Inversion | Translocation | Duplication | Insertion | Deletion | HDR[b] |
| 1 | 228584 | 18921 | 15759 | 1192 | 16899 | 14385 | 1195 |
| 2 | 249810 | 17848 | 17237 | 1309 | 18989 | 16601 | 1276 |
| 3 | 313984 | 24697 | 21104 | 2192 | 22431 | 21298 | 1691 |
| 4 | 248792 | 20975 | 15305 | 1394 | 18125 | 14582 | 1190 |
| 5 | 259863 | 21183 | 19078 | 1985 | 20820 | 16103 | 1265 |
| 6 | 249875 | 20807 | 17953 | 1052 | 18643 | 14204 | 1389 |
| 7 | 253439 | 20970 | 18502 | 1224 | 17056 | 17257 | 1295 |
| 8 | 205584 | 19999 | 16605 | 1264 | 17102 | 14904 | 1287 |
| 9 | 235807 | 19836 | 17111 | 1287 | 17280 | 17765 | 1309 |
| *C. glauca* | | | | | | | |
| Chromosome# | SNP[a] | Inversion | Translocation | Duplication | Insertion | Deletion | HDR[b] |

| | | | | | | | |
|---|---|---|---|---|---|---|---|
| 1 | 225389 | 21799 | 15100 | 939 | 15918 | 13949 | 916 |
| 2 | 246615 | 20727 | 16558 | 1056 | 18005 | 16165 | 997 |
| 3 | 310789 | 27576 | 20645 | 1939 | 21450 | 20859 | 1412 |
| 4 | 245597 | 23854 | 14676 | 1135 | 17144 | 14145 | 911 |
| 5 | 256668 | 24061 | 18420 | 1732 | 19839 | 15671 | 986 |
| 6 | 246680 | 23685 | 17294 | 797 | 17662 | 13765 | 1110 |
| 7 | 250242 | 23848 | 17843 | 971 | 16075 | 16818 | 1016 |
| 8 | 202389 | 22878 | 15946 | 1011 | 16121 | 14466 | 1008 |
| 9 | 232612 | 22715 | 16452 | 1034 | 16299 | 17329 | 1030 |

[a] single nucleotide polymorphism
[b] highly diverged regions

**Supplementary Table S4.** Orthogroup statistics of nine citrus species, including four Australian limes.

| Citrus species | *C. australasica* | *C. inodora* | *C. glauca* | *C. australis* | *C. clementina* | *C. limon* | *C. maxima* | *C. sinensis* | *C. trifoliata* |
|---|---|---|---|---|---|---|---|---|---|
| Total Genes | 27,348 | 28,173 | 30,066 | 29,464 | 24,533 | 32,894 | 30,123 | 29,138 | 25,537 |
| Number of genes in orthogroups | 26,864 (98.2%) | 27494 (97.6%) | 29,351 (97.6%) | 28,375 (96.3%) | 23,884 (97.4%) | 27,355 (83.2%) | 28,725 (95.4%) | 27,315 (93.7%) | 25,268 (98.9%) |
| Number of species-specific orthogroups | 91 | 105 | 155 | 97 | 17 | 574 | 223 | 220 | 42 |
| Number of genes in species-specific orthogroups | 1,372 (5.0%) | 286 (1.0%) | 1,421 (4.7%) | 1,941 (6.6%) | 43 (0.2%) | 1,325 (4.0%) | 789 (2.6%) | 1,193 (4.1%) | 135 (0.5%) |
| Number of unassigned genes (singletons) | 484 (1.8%) | 679 (2.4%) | 715 (2.4%) | 1,089 (3.7%) | 649 (2.6%) | 5,539 (16.8%) | 1,398 (4.6%) | 1,823 (6.3%) | 269 (1.1%) |
| Total species-specific genes | 1,856 | 965 | 2,136 | 3,030 | 692 | 6,864 | 2,187 | 3,016 | 404 |

**Supplementary Table S5.** List of samples used for transcriptome analysis and read mapping yields.

| Number | Species | Tissue type | Yield (Mb) | Number of reads (million) (Q ≥ 30) |
|---|---|---|---|---|
| 1 | *C. australasica* | Flowers, buds, and just-formed fruit | 11.96 | 79.7 |
| 2 | *C. australasica* | Mature leaves | 11.64 | 77.6 |
| 3 | *C. australasica* | Bark | 10.6 | 70.7 |
| 4 | *C. australasica* | Fruit 0.5-1 inches long | 10.01 | 66.8 |
| 5 | *C. australasica* | Mature leaves | 10.53 | 70.2 |
| 6 | *C. australasica* | Fruit - 1.5-2 inches long | 10.77 | 71.8 |
| 7 | *C. australasica* | Bark from mature stem | 10.19 | 68.0 |
| 8 | *C. australasica* | Young stems (whole, with thorns) | 12.13 | 80.9 |
| 9 | *C. inodora* | Young shoots with tender leaves | 9.78 | 65.2 |
| 10 | *C. inodora* | Mature leaves | 10.17 | 67.8 |
| 11 | *C. inodora* | Bark | 10.54 | 70.3 |
| 12 | *C. inodora* | Flowers, buds | 10.38 | 69.2 |
| 13 | *C. inodora* | Mature leaves | 10.77 | 70.8 |
| 14 | *C. inodora* | Bark | 10.62 | 78.6 |
| 15 | *C. glauca* | Young shoots with tender leaves | 11.79 | 78.6 |
| 16 | *C. glauca* | Mature leaves | 11.43 | 76.2 |
| 17 | *C. glauca* | Bark | 11.04 | 73.6 |
| 18 | *C. glauca* | Flowers, buds, very young fruit | 12.1 | 80.7 |
| 19 | *C. glauca* | Mature leaves | 11.81 | 78.7 |

| | | | | |
|---|---|---|---|---|
| 20 | *C. glauca* | Stem pieces | 12.35 | 82.3 |

**Supplementary Table S6.** Summary of *de novo* transcriptome assembly of quality filtered paired-end RNA-seq reads of three Australian lime species.

| Statistics | *C. australasica* | *C. inodora* | *C. glauca* |
|---|---|---|---|
| No. of Contigs | 282,079 | 252,646 | 283,961 |
| No. of Contigs (>=1000 bp) | 146,441 | 138,788 | 139,222 |
| No. of of Contigs (>=10000 bp) | 2,257 | 1,747 | 2,019 |
| Largest Contig (bp) | 21,050 | 22,261 | 21,989 |
| Total Length (bp) | 549,752,050 | 501,589,743 | 520,340,891 |
| Mean Length (bp) | 1,948.93 | 1,985.35 | 1,832.44 |
| N50 | 3,772 | 3,641 | 3,654 |
| N70 | 2,455 | 2,404 | 2,385 |
| N90 | 923 | 984 | 848 |
| GC (%) | 38.17 | 38.39 | 38.79 |

**Supplementary Table S7.** Number of genes and transcripts predicted in each chromosome of the primary and alternate haplotypes.

| Genome assembly | | *C. australasica* | | *C. inodora* | | *C. glauca* | |
|---|---|---|---|---|---|---|---|
| Haplotype | Chromosome # | Genes | Transcripts | Genes | Transcripts | Genes | Transcripts |
| Primary | 1 | 2,585 | 2,847 | 2,980 | 3,364 | 2,927 | 3,285 |
| | 2 | 3,108 | 3,421 | 3,675 | 4,153 | 3,535 | 3,928 |
| | 3 | 4,299 | 4,764 | 4,446 | 4,993 | 5,015 | 5,578 |
| | 4 | 3,169 | 3,476 | 3,176 | 3,589 | 2,896 | 3,248 |
| | 5 | 2,795 | 3,079 | 2,946 | 3,269 | 3,029 | 3,427 |
| | 6 | 2,418 | 2,700 | 2,750 | 3,071 | 2,736 | 3,087 |
| | 7 | 2,396 | 2,658 | 2,773 | 3,086 | 2,549 | 2,831 |
| | 8 | 2,385 | 2,679 | 2,708 | 3,035 | 2,576 | 2,886 |
| | 9 | 2,260 | 2,501 | 2,722 | 3,072 | 2,151 | 2,410 |
| Alternate | 1 | 2,534 | 2,825 | 2,925 | 3,318 | 3,107 | 3,488 |
| | 2 | 3,109 | 3,464 | 3,563 | 4,014 | 3,715 | 4,151 |
| | 3 | 4,080 | 4,491 | 4,400 | 4,983 | 5,118 | 5,691 |
| | 4 | 2,559 | 2,792 | 3,196 | 3,637 | 2,890 | 3,211 |
| | 5 | 2,682 | 2,967 | 2,915 | 3,300 | 3,029 | 3,440 |
| | 6 | 2,284 | 2,558 | 2,771 | 3,126 | 2,954 | 3,306 |
| | 7 | 2,337 | 2,582 | 2,675 | 2,999 | 2,649 | 2,935 |
| | 8 | 2,373 | 2,671 | 2,708 | 3,039 | 2,496 | 2,792 |
| | 9 | 2,298 | 2,567 | 2,512 | 2,829 | 2,186 | 2,443 |

**Supplementary Table S8.** Completeness assessment of de novo assemblies of three Australian lime species

| *BUSCO Notation | *C. australasica* | *C. inodora* | *C. glauca* |
|---|---|---|---|
| Complete single copy | 22 (5.18%) | 25 (5.88%) | 15 (3.53%) |
| Complete duplicated | 376 (88.47%) | 372 (87.53%) | 390 (91.76%) |
| Fragmented | 26 (6.12%) | 25 (5.88%) | 19 (4.47%) |
| Missing | 1 (0.24%) | 3 (0.71%) | 1 (0.24%) |

* BUSCO analysis was conducted with BLAST cutoff e-value of $1x10^{-3}$ using a BUSCO dataset (n=425) for Viridiplantae.

**Supplementary Table S9.** Summary of ORF prediction followed by GO annotation of de novo assembly of three Australian lime species.

| Procedure (tool) | Outcome | *C. australasica* | *C. inodora* | *C. glauca* |
|---|---|---|---|---|
| *de novo* assembly (Trinity v2.5) | Total number of *de novo* contigs | 282,079 | 252,646 | 283,961 |
| sequence clustering (CD-HIT) | Number of *de novo* contigs after removing redundant contigs | 186,986 | 217,158 | 221,159 |
| ORF prediction (TransDecoder 5.5.0) | ORF type | | | |
| | No. of complete ORFs | 107,562 (76.41%) | 122,526 (73.47%) | 113,069 (75.44%) |
| | No. of 5' partial ORFs (missing start codon) | 160,10 (11.37%) | 18,550 (11.12%) | 17,304 (11.54%) |
| | No. of 3' partial ORFs (missing stop codon) | 10,717 (7.61%) | 13,818 (8.29%) | 10,891 (7.27%) |
| | No. of internal ORFs (missing start and stop codon) | 6,489 (4.61%) | 11,871 (7.12%) | 8,621 (5.75%) |
| | Total number of predicted ORFs | 140,778 | 166,765 | 149,885 |
| GO annotation (OmicsBox 3.0.30) | No. of ORFs with Blastx hits | 101,103 | 113,587 | 106,178 |
| | No. of ORFs with GO mapping | 100,649 | 113,083 | 105,776 |
| | No. of annotated ORFs | 92,278 | 103,232 | 97,421 |
| | % of annotated ORFs | 65.55% | 61.9% | 65% |

**Supplementary Table S10.** Number of annotated sequences in the de novo assemblies of three Australian lime species.

| Species | No. of annotated sequences | Number of sequences | | |
|---|---|---|---|---|
| | | Biological Process (GO:0008150) | Cellular Component (GO:0005527) | Molecular Function (GO:0003674) |
| *C. australasica* | 92,278 | 80,848 | 77,477 | 81,160 |
| *C. inodora* | 103,232 | 90,451 | 87,481 | 90,748 |
| *C. glauca* | 97,421 | 85,341 | 82,767 | 85,559 |

**Supplementary Table S11.** Functional annotation of a subset of genes that are involved in Innate immune response (GO:0045087).

| GO term (GO ID) | Number of sequences | | |
|---|---|---|---|
| | *C. australasica* | *C. inodora* | *C. glauca* |
| Biological process (GO:0008150) | 80840 | 90451 | 85341 |
| Response to other organism (GO:0009725) | 10069 (12.46%) | 10663 (11.76%) | 10380 (12.16%) |
| Defense response to other organism (GO:0098542) | 8232 (81.76%) | 8624 (81.11%) | 8587 (82.73%) |
| Innate immune response (GO:0045087) | 2769 (33.64%) | 2957 (34.29%) | 2802 (26.99%) |
| plant-type hypersensitive response (GO:0009626) | 971 (35.07%) | 896 (30.3%) | 839 (29.94%) |
| pattern recognition receptor signaling pathway (GO:000221) | 418 (15.1%) | 412 (13.93%) | 422 (15.06%) |
| Cell surface pattern recognition receptor signaling pathway (GO:0002752) | 46 (11%) | 63 (15.29%) | 60 (14.22%) |
| pathogen-associated molecular pattern receptor signaling pathway (GO:0140426) | 128 (30.62%) | 95 (23.06%) | 98 (23.22%) |

**Supplementary Table S12.** Genome size comparison of cultivated citrus species and the Australian limes.

| Species and genome version | Haplotype | Assembly size (Mb) | Reference |
|---|---|---|---|
| *Citrus australasica* v1.0 | Primary | 336.7 | Present study |
| | Alternate | 335.2 | |
| *C.inodora* v1.0 | Primary | 303.7 | Present study |
| | Alternate | 298.8 | |
| *C. glauca* v1.0 | Primary | 376.4 | Present study |
| | Alternate | 379.2 | |
| *C. limon* L. Burm f. v1.0 | Primary | 312.8 | Guardo et al. 2021 |
| | Alternate | 324.74 | |
| *C. limon* cv. Eureka v1.0 | | 316 | Bao et al. 2023 |
| *C. limon* cv. Xiangshui genome v1.0 | | 365 | Yu et al. 2024 |
| *C. australis* v1.0 | | 331 | Nakandala et al., 2023 |
| *C. sinensis* | | 319-328 | Xu et al. 2013 & Wu et al. 2014 |
| *C. clementina* | | 301.4 | Wu et al. 2014 |
| *C. reticulata* | | 334 | Wang et al. 2018 |
| *C. ichangensis* | | 357 | Wang et al. 2017 |
| *C. maxima* (C. grandis) v1.0 | | 345 | Wang et al. 2017 |
| *C. maxima* Cupi Majiayou v1.0 | | 368 | Lu et al. 2022 |
| *C. medica* v1.0 | | 406 | Wang et al. 2017 |
| *C. reticulata* v1.0 | | 347 | Wang et al. 2018 |
| *C. sinensis*Valencia v2.0 | | 338 | Wang et al. 2021 |
| *C. sinensis*Di-Haploid Sweet Orange (DHSO) v3.0 | | 337 | Wang et al. 2021 |
| *Atalantia buxifolia* v1.0 | | 316 | Wang et al. 2017 |
| *Poncirus trifoliata* v1.3.1 | | 265 | Peng et al. 2020 |

**Supplementary Table S13.** Summary of Hi-C analysis for generating chromosome-scale scaffolds of *Citrus australasica*, *C. inodora,* and *C. glauca*.

| Sample Name | Raw reads (bp) | Chromosome-scale scaffolds | Length of Sequence (bp) | % of corrected contig assembly[a] | % of 9 large scaffolds[b] |
|---|---|---|---|---|---|
| *Citrus australasica* | 171,943,547 | 9 | 316,870,332 | 91.03 | 86.1 (P), 86.2 (A) |
| *C. inodora* | 130,774,438 | 9 | 293,498,500 | 92.56 | 91.2 (P), 91.03 (A) |
| *C. glauca* | 155,778,564 | 9 | 341,731,096 | 90.8 | 91.5 (P, 91.02 (A) |

[a] percentage of the total length of the contigs that were involved in clusters.
[b] percentage nine large scaffold of primary (P) and alternate (A) haplotype after chromosome scale-scaffolding.
